# Genomics of cold adaptations in the Antarctic notothenioid fish radiation

**DOI:** 10.1038/s41467-023-38567-6

**Published:** 2023-06-09

**Authors:** Iliana Bista, Jonathan M. D. Wood, Thomas Desvignes, Shane A. McCarthy, Michael Matschiner, Zemin Ning, Alan Tracey, James Torrance, Ying Sims, William Chow, Michelle Smith, Karen Oliver, Leanne Haggerty, Walter Salzburger, John H. Postlethwait, Kerstin Howe, Melody S. Clark, H. William Detrich, C.-H. Christina Cheng, Eric A. Miska, Richard Durbin

**Affiliations:** 1 Wellcome Sanger Institute, Tree of Life, Wellcome Genome Campus, Hinxton, CB10 1SA UK; 2 Department of Genetics, University of Cambridge, Downing Street, Cambridge, CB2 3EH UK; 3 Wellcome/CRUK Gurdon Institute, University of Cambridge, Tennis Court Rd, Cambridge, CB2 1QN UK; 4 Naturalis Biodiversity Center, Leiden, 2333 CR the Netherlands; 5 University of Oregon, Institute of Neuroscience, 1254 University of Oregon, 13th Avenue, Eugene, OR 97403 USA; 6 University of Oslo, Natural History Museum, University of Oslo, Sars’ gate 1, 0562 Oslo, Norway; 7 University of Zurich, Department of Palaeontology and Museum, University of Zurich, Karl-Schmid-Strasse 4, 8006 Zurich, Switzerland; 8 European Molecular Biology Laboratory, European Bioinformatics Institute, Wellcome Genome Campus, Hinxton, CB10 1SA UK; 9 University of Basel, Zoological Institute, Department of Environmental Sciences, Vesalgasse 1, 4051 Basel, Switzerland; 10 British Antarctic Survey, High Cross, Madingley Road, Cambridge, CB3 0ET UK; 11 Northeastern University, Department of Marine and Environmental Sciences, Marine Science Centre, 430 Nahant Rd., Nahant, MA 01908 USA; 12 Department of Evolution, Ecology, and Behaviour, University of Illinois, Urbana-Champaign, IL 61801 USA

**Keywords:** Comparative genomics, Adaptive radiation, Evolutionary genetics, Evolutionary biology

## Abstract

Numerous novel adaptations characterise the radiation of notothenioids, the dominant fish group in the freezing seas of the Southern Ocean. To improve understanding of the evolution of this iconic fish group, here we generate and analyse new genome assemblies for 24 species covering all major subgroups of the radiation, including five long-read assemblies. We present a new estimate for the onset of the radiation at 10.7 million years ago, based on a time-calibrated phylogeny derived from genome-wide sequence data. We identify a two-fold variation in genome size, driven by expansion of multiple transposable element families, and use the long-read data to reconstruct two evolutionarily important, highly repetitive gene family loci. First, we present the most complete reconstruction to date of the antifreeze glycoprotein gene family, whose emergence enabled survival in sub-zero temperatures, showing the expansion of the antifreeze gene locus from the ancestral to the derived state. Second, we trace the loss of haemoglobin genes in icefishes, the only vertebrates lacking functional haemoglobins, through complete reconstruction of the two haemoglobin gene clusters across notothenioid families. Both the haemoglobin and antifreeze genomic loci are characterised by multiple transposon expansions that may have driven the evolutionary history of these genes.

## Abstract (web summary)

The notothenioid radiation is a remarkable group of fish adapted to life in the icy waters of the Southern Ocean. This study investigates the evolutionary history of this group and the basis of their adaption to cold environments through genomic analysis of 24 new genome assemblies.

## Introduction

**Fig. 1:**
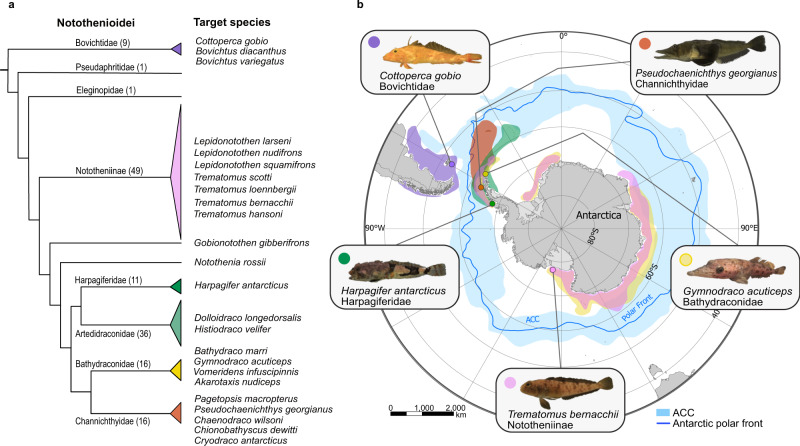
The Notothenioidei suborder, all 24 target species, and representative geographic distributions of PacBio sequenced species. **a** Notothenioid families and a number of species contained in each family based on the species list by ref. ^3^ are shown in parentheses, except for the Nototheniidae, which are paraphyletic, containing the Nototheniinae, *G. gibberifrons* and *N. rossi*. The target species sequenced in the present study are listed next to the tree. **b** Map of Antarctica and the Southern Ocean showing the geographic distribution of the five notothenioid species sequenced with PacBio. Colours correspond to the respective families on the tree. Coloured circles on the map indicate the sampling location. The blue belt around Antarctica indicates the region of the Antarctic Circumpolar Current (ACC), and the thin blue line the Antarctic polar front. The map was generated using ArcGIS (GIS Software, Version 10.0). Data for the geographic distribution of each species were derived from the Scientific Committee for Antarctic Research (SCAR), Antarctic Biodiversity Portal (https://www.biodiversity.aq/), comprising occurrence records from multiple databases. Source data are provided as a Source Data file.

The suborder Notothenioidei is a textbook example of a marine fish adaptive radiation, with notothenioids being the dominant fish group of the Southern Ocean, both in terms of species richness and biomass, comprising between 130–140 species^1–3^ (Fig. 1a). The establishment and initial diversification of the notothenioids is closely linked to the separation of the Antarctic continent from surrounding land masses and the subsequent establishment of the Antarctic Circumpolar Current (ACC)^4^ (Fig. 1b). Formation of the ACC exacerbated the isolation of the Antarctic continent and contributed to cooling of the surrounding waters, glaciation of the continent, and appearance of sea ice^5^. These events in turn extirpated most of the original temperate fish fauna, and notothenioids expanded to fill the abandoned niches as they evolved adaptations to life in the isolated, cold, and highly oxygenated waters of the Southern Ocean^6,7^. Since notothenioids include species occurring in cool-temperate non-Antarctic regions^8^, as well as species occurring in icy, freezing higher latitudes (known as the “Antarctic clade” or Cryonotothenioidea (cryonotothenioids))^9^, they represent a powerful model for the study of the genomic origins of extremophiles. Adaptations to cold include the presence of antifreeze glycoproteins (AFGPs)^10^, the lack of the classic heat shock response^11^, and the presence of giant muscle fibres in some notothenioids^12^. Further, a striking respiratory phenotype arose in the derived family Channichthyidae (“icefishes”), including the complete loss of functional haemoglobins in all of its species and the loss of cardiac myoglobin in six of them^13^. While haemoglobin loss was not lethal in the oxygenated waters of the cold Southern Ocean, these losses were likely not without fitness consequences, as indicated by numerous compensatory cardiovascular adaptations, including enlarged hearts, and increased vascular bores^4^.

The in-depth characterisation of notothenioid genomes has been hampered in the past by their complex genome characteristics, such as high levels of repeats and heterozygosity, that have hindered the accuracy of genome assemblies based on short-read data. Furthermore, the few available high-quality notothenioid genome assemblies^14,15^ cover only a small portion of the diversity in this group. The Vertebrate Genomes Project (VGP) (https://vertebrategenomesproject.org/)^16^ has demonstrated that long-read sequencing technologies can generate highly contiguous genome assemblies even for the most technically difficult species.

In this work, we achieve a step-change increase in Antarctic notothenioid genome resources for the broader community. We apply the VGP pipeline and standards to five selected notothenioid species representing key points in the radiation, and use other sequencing approaches, such as Illumina and linked reads to generate a total of 24 new genomes. Collectively these assemblies cover six of the eight notothenioid families (all except two non-Antarctic single-species families) (Fig. 1a), including the five families that comprise the Antarctic radiation, and a non-Antarctic family. We use these new genome assemblies to address previously unresolved questions about the evolutionary history of the radiation. First, we present a new time-calibrated phylogeny, and with it a new time estimate for the expansion of the radiation. Next, we identify a significant genome size expansion and investigate the role of transposable elements (TEs) in cold adaptation throughout this adaptive radiation. Finally, we investigate the evolutionary history of two adaptively important gene families, the antifreeze genes, which were essential for survival in icy waters, and the haemoglobin genes, which were ultimately lost in icefish, the only vertebrate lacking functional haemoglobins.

## Results and discussion

### Genome sequencing, assembly, and annotation

We generated and analysed reference genome assemblies for 24 notothenioid fish species across the radiation using a variety of sequencing technologies (Fig. 1, Table 1, Supplementary Data 1). Genomes of five species — *Cottoperca gobio* (synonymised by many to *Cottoperca trigloides*^3^)*, Trematomus bernacchii, Harpagifer antarcticus, Gymnodraco acuticeps, Pseudochaenichthys georgianus* — each representing a different notothenioid family, were assembled using Pacific Biosciences (PacBio) long reads, in combination with 10X Genomics linked reads and Hi-C data (Methods). Briefly, we used Falcon-unzip^17^ to generate each primary assembly from the PacBio reads and then applied 10X Genomics Chromium data for scaffolding and polishing. The genomes of *C. gobio*, *H. antarcticus* and *P. georgianus* were further scaffolded using Bionano hybrid scaffolding. In addition, for *C. gobio* and *P. georgianus* we also used Hi-C data (ARIMA and Dovetail respectively) for scaffolding with SALSA2^18^. To further improve the quality of these genomes, we performed manual curation using the Genome Evaluation Browser (gEVAL)^19^ to remove mis-assemblies, false duplications, and sequencing contaminations such as symbionts and adapter sequences, and to merge scaffolds based on supporting evidence^20^ (Supplementary Data 2). The genomes of *C. gobio* and *P. georgianus*, were assigned to 24 chromosomes, consistent with their known karyotypes^21,22^ (Supplementary Fig. 1a–b), with scaffold N50 25 Mb and 43 Mb, respectively. The reference assembly for *C. gobio* (fCotGob3.1, GCA_900634415.1) was previously described in ref. ^23^. The other PacBio genomes were assembled to scaffolds, *T. bernacchii* (N50 8.8 Mb), *H. antarcticus* (N50 5.0 Mb), and *G. acuticeps* (N50 1.9 Mb). Genomes of 11 more species were sequenced with 10X Genomics using a single linked-read library for each and assembled with Supernova v2.0^24^ with an average N50 of 2.6 Mb **(**Supplementary Data 2). Genomes of eight additional species were sequenced using only Illumina HiSeq reads and assembled using a reference-guided approach. For these eight species, a primary assembly was generated with SOAPdenovo2^25^, and scaffolding was done using the closest PacBio genome as reference (except for *Bathydraco marri* for which scaffolding with a reference assembly failed) (Methods, Supplementary Data 3). This approach generally improved the N50 and BUSCO completeness for these species compared to the primary assembly. All 19 assemblies were also manually curated to remove external contamination, and false duplications (the latter in Supernova assemblies). We observed a smaller genome size in short read assemblies, compared to the PacBio and linked read ones from the same families (Supplementary Data 2). We attribute this to short read assemblers tending to collapse repeat regions during the assembly process^16^^.^

**Table 1:** Target species, data types, and assembly statistics

Family	Species	ID	Data type	Size (Mb)	Het. (%)	Scaff. count	Scaff.N50 (Mb)	BUSCO (%)
Bovichtidae	*Cottoperca gobio (Cottoperca trigloides)*	fCotGob3	PB, 10X, BN, HiC	609	0.31	322	25.156	93.4
	*Bovichtus diacanthus*	fBovDia2	10XG	641	0.49	12,443	7.334	92.7
	*Bovichtus variegatus*	fBovVar2	10XG	657	0.58	11,172	14.072	92.8
Nototheniidae	*Trematomus bernacchii*	fTreBer1	PB, 10XG	867	0.48	864	8.748	97.5
	*Gobionotothen gibberifrons*	fGobGib1	10XG	784	0.57	46,943	0.848	80.7
	*Lepidonotothen larseni (Nototheniops larseni)*	fLepLar1	HiSeqX	839	0.69	841,998	0.004	43.9
	*Lepidonotothen nudifrons (Nototheniops nudifrons)*	fLepNud1	10XG	728	0.34	29,926	1.385	88.1
	*Lepidonotothen squamifrons*	fLepSqu1	HiSeqX	758	0.48	478,411	0.009	63.6
	*Notothenia rossii*	fNotRos1	10XG	988	0.76	50,621	1.691	85.2
	*Trematomus hansoni*	fTreHan1	HiSeqX	708	0.44	178,347	0.047	68.8
	*Trematomus loennbergii*	fTreLoe1	10XG	801	0.63	48,076	1.316	84.3
	*Trematomus scotti*	fTreSco1	HiSeqX	832	0.62	696,803	0.004	47.3
Harpagiferidae	*Harpagifer antarcticus*	fHarAnt1	PB, 10XG, BN	941	0.48	1541	4.997	94.1
Artedidraconidae	*Histiodraco velifer*	fHisVel1	10XG	831	0.33	62,325	0.059	82.4
	*Dolloidraco longedorsalis*	fDolLon1	HiSeqX	846	0.49	492,743	0.037	58.8
Bathydraconidae	*Gymnodraco acuticeps*	fGymAcu1	PB, 10XG	997	0.54	2618	1.921	93.5
	*Akarotaxis nudiceps*	fAkaNud1	10XG	844	1.07	83,165	0.067	75.2
	*Bathydraco marri*	fBatMar1	HiSeqX	890	0.53	688,793	0.005	52.6
	*Vomeridens infuscipinnis*	fVomInf1	HiSeqX	833	0.38	507,682	0.008	68.6
Channichthyidae	*Pseudochaenichthys georgianus*	fPseGeo1	PB, 10XG, HiC	1,026	0.24	1563	42.832	94.1
	*Chaenodraco wilsoni*	fChaWil1	10XG	956	1.00	61,567	0.943	80.0
	*Chionobathyscus dewitti*	fChiDew1	HiSeqX	900	0.4	555,224	0.006	64.5
	*Cryodraco antarcticus (Pagetodes antarcticus)*	fCryAnt1	10XG	944	0.6	46,843	0.809	82.5
	*Pagetopsis macropterus*	fPagMac1	10XG	958	0.6	58,353	0.485	80.5

Data types used to generate assemblies: PB: PacBio CLR, 10XG: 10X Genomics linked reads, BN: Bionano, HiSeqX: Illumina Hi-Seq, HiC: Hi-C from ARIMA (*C. gobio*), Dovetail (*P. georgianus*). Gene completeness was calculated with BUSCO v.5, and heterozygosity (Het.) estimated with GenomeScope (kmer = 31). Names in parentheses are recent nomenclature revisions^3^. Scaff = scaffold. Additional information regarding samples and assembly quality for all species is provided in Supplementary Data 1 and 2.

For the PacBio assemblies, BUSCO^26^ gene completeness averages 95% (Supplementary Fig. 1c), for 10X assemblies BUSCO averages 86%, and for short read assemblies 65%. Gene prediction for all PacBio assemblies was performed via the Ensembl Gene Annotation system^27^ (Methods). The BUSCO completeness of the gene annotation averages 92% (Supplementary Fig. 1d). Approximately 23–24,000 genes were annotated for the four cryonotothenioid species (24,373 for *T. bernacchii*, 23,146 for *H. antarcticus*, 24,091 for *G. acuticeps*, and 23,222 for *P. georgianus* (Supplementary Data 4)), around 2000 genes more than the non-Antarctic species *C. gobio* (Ensembl genes: 21,662^23^), suggesting that cold adaptation was accompanied by an expansion in the number of genes in notothenioids (Supplementary Fig. 1e–f).

### A new time-calibrated phylogeny for notothenioids

**Fig. 2:**
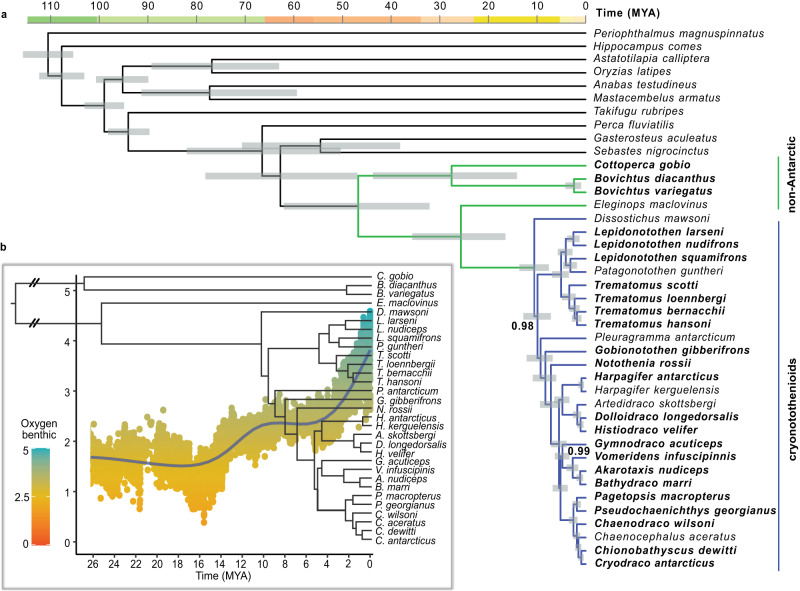
New time-calibrated phylogeny and paleoclimate analysis. **a** Time-calibrated phylogeny of 41 percomorph fish species, including 31 species of notothenioids and 10 outgroup fish species, generated with BEAST2^39^. Branch length corresponds to time in million years (MYA) and grey rectangles show 95% highest posterior density intervals for node age estimates. All nodes received full support (Bayesian posterior probability = 1) except where noted. Species in bold were sequenced in this study. Branches for the Antarctic clade are highlighted in blue (cryonotothenioids), and non-Antarctic notothenioid species are marked in green. **b** Diversification of notothenioid species and temperature variation through time. Tree based on notothenioid species from panel a. The scatterplot shows data based on deep-sea δ^18^O benthic records which inversely reflect temperature with higher δ^18^O benthic corresponding to lower temperatures (green) and lower δ^18^O corresponding to higher temperatures (orange). The oxygen benthic is expressed as a ratio of two concentrations of oxygen isotopes^36^; blue line shows moving average (Generalised Additive Model). Source data can be found in the Dryad repository at 10.5061/dryad.80gb5mktn.

Our multi-species dataset affords the opportunity to establish a new time-calibrated phylogeny for the notothenioid radiation based on genome-wide data, to help resolve controversies about the timing of the diversification of the notothenioids relative to the chilling of the Southern Ocean. Most of the previously published phylogenetic hypotheses for notothenioids were based on limited numbers of genes^6^, RAD-seq^28,29^, or exome capture data^30^. By analysing genome-wide data extracted from BUSCO single copy genes from 41 percomorph fish species, including the 24 new and eight previously published notothenioid genomes^31,32^ we provide the most comprehensive phylogenomic analyses of notothenioids to date, with taxonomic coverage of most of their sub-groups. Based on this analysis, calibrated using established teleost divergence dates^33^, the onset of diversification of the Cryonotothenioidea, which are characterised by the presence of AFGPs, is estimated at around 10.7 million years ago (MYA) (highest posterior density interval: 14.1–7.8 MYA) (Fig. 2a). Previous work estimated this time at ~22 MYA^6,34^. While the appearance of AFGPs was previously estimated at 42–22 MYA, which would predate the major cooling of the Southern Ocean^6^, our new analysis indicates that the emergence of AFGPs occurred between 26.3–10.7 MYA. This period includes the Middle Miocene Climate Transition at 15–13 MYA and the subsequently increased Antarctic glaciation^35^. Furthermore, our analysis highlights that many speciation events in each major family took place within the last 5 million years (Fig. 2a).

To investigate climatic events that might have driven diversification in derived notothenioid clades we examined paleoclimate data, represented by δ^18^O records (derived from the ratio of ^18^O/^16^O stable isotopes), which reflect variations in the temperature of seawater^36^. These data indicate substantial fluctuations in global mean sea levels (GMSL) during the early Pliocene, and a sustained temperature drop in Antarctica ~3 MYA, which led to the rapid formation of large sea ice sheets^36^. Variations in sea ice formation may have played an important role in isolating populations, leading to further diversification of the cryonotothenioids (Fig. 2b). A similar influence of cooling events has been suggested in other non-Antarctic cold-adapted radiations (e.g. aquatic crustacea and orchids)^37,38^, where diversification has been linked to past changes in global temperature.

Furthermore, our BEAST2^39^ analyses support the monophyly of dragonfishes (family Bathydraconidae, represented here by *Vomeridens infuscipinnis, Akarotaxis nudiceps, Bathydraco marri*, and *G. acuticeps*), as indicated by morphology and by RADseq data^29^, while other methods and previous studies^30,40^ suggested that they are paraphyletic. This result was also found with maximum likelihood phylogeny reconstruction using IQ-TREE^41^ when using the “strict” set of alignments (see Methods for definition). However, we note that when using the”permissive” or full alignment sets, concatenated IQ-TREE found the alternative, paraphyletic phylogeny that places *G. acuticeps* with the Channnichthyidae, albeit with relatively weak bootstrap values of 82 and 70, respectively. All notothenioid nodes apart from this node were found in all analyses, and had bootstrap values of at least 97.0. ASTRAL^42^ analysis of the IQTREE gene trees also place *G. acuticeps* with the Channnichthyidae with Bayesian posterior probability from 0.38 to 0.62 depending on the filtering level (see Methods). In contrast to previous studies^29,34^, none of our phylogenetic analyses group together the neutrally buoyant *Pleuragramma antarcticum* and *Dissostichus* spp. We therefore suggest that neutral buoyancy evolved independently in these two lineages.

### Transposon expansion is driving genome size evolution in notothenioids

Transposable element dynamics are increasingly recognised as major drivers of evolutionary innovation, and their analysis is greatly facilitated by the use of long-read sequencing technologies^43,44^. For example, the location of TE insertions can influence the expression of nearby genes and induce phenotypic variation^45,46^. The diversity of transposons varies substantially between organisms, with teleost fish genomes containing greater TE diversity compared to other vertebrates, such as mammals^43,47^. In teleosts, genome size tends to correlate with transposon abundance, while a reduction in genome size does not necessarily correspond to lower transposon diversity, but is more commonly caused by reduced copy numbers of TEs^48,49^. Here, we use a set of long read and linked read assemblies, together with high-quality de novo annotations, to investigate the expansions of transposable elements in notothenioids in relation to their genome sizes. We also investigate the timing of these expansions with respect to major lineage diversification events in the radiation.

**Fig. 3:**
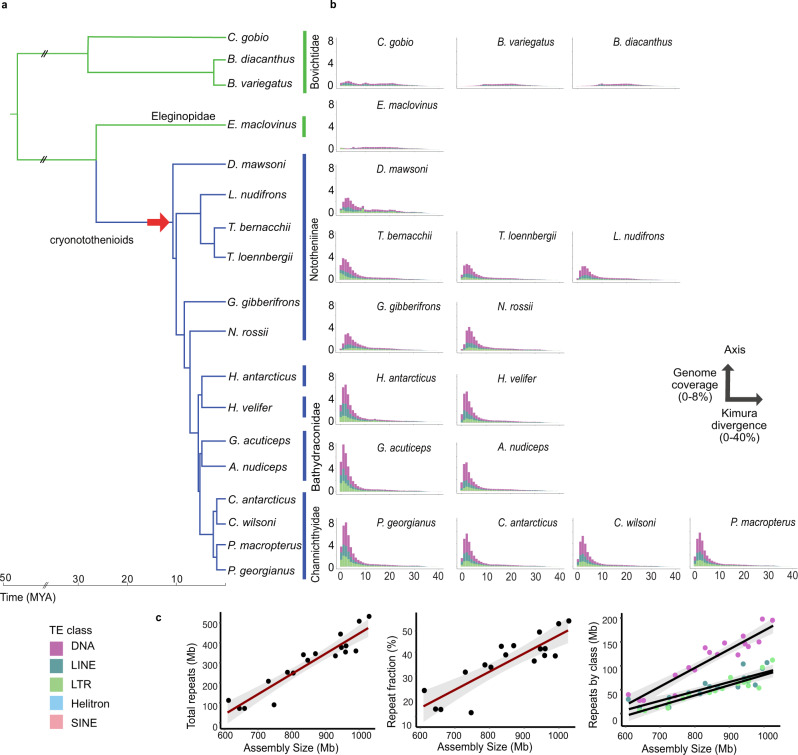
Expansion of transposable elements and genome size variation in notothenioid genomes. **a** Species analysed, including 16 species sequenced in this study, and two previously published genomes (*E. maclovinus*^32^, and *D. mawsoni*^57^). **b** Repeat landscape plots showing the distribution of transposable element copies as percentage of divergence from consensus repeat models (*x*-axis, Kimura divergence) versus genome coverage (*y*-axis). Colours represent different TE classes. The red arrow indicates the timing of the earliest TE expansion identified in our analysis. **c** Correlation of repeat content with genome size (Pearson Correlation Coefficient, *n* = 16, *R* = 0.95, two-sided *p* = 1.647e-08, slope = 0.99), an increase of repeat fraction with genome size, and increase of DNA, LINE, and LTR TE classes with genome size. The shaded zone indicates 95% confidence interval. The plot was generated using package ggplot2 and function ggpubr^99^. Double forward slashes in the time axis indicate a cropped line in the tree branches. Source data are provided as a Source Data file.

We identified substantial variation in assembled genome size across the notothenioid phylogeny with the smallest genomes identified in the non-Antarctic temperate-water family Bovichtidae (0.6 Gb), which form a sister group to all other extant notothenioids, and the largest genomes in the high-latitude icefish species of the derived family Channichthyidae (1.1 Gb) (Fig. 3, Table 1, Supplementary Data 2). This observation is consistent with earlier estimates of large genome sizes in icefish based on flow cytometry^50^. The variation in genome size is nearly completely accounted for by changes in the total repeat content, suggesting that it is driven by TE expansions (Fig. 3c). Such expansions are found in diverse members of the Antarctic cryonotothenioids, including *Dissostichus*, the sister lineage to all other cryonotothenioids, indicating that the onset of TE expansion was associated with the radiation of the clade (Fig. 3a, b). This potentially indicates that the onset of the TE expansion coincided with, or possibly predated, the first diversification event in cryonotothenioids. TE expansion continued in the more derived clades (e.g., dragonfishes and icefishes), consistent with lineage-dependent expansion characterised by multiple young insertions as seen in a TE landscape analysis (Fig. 3b). Further, we found that this increase in TE content is due to the simultaneous amplification of multiple TE families, including both DNA transposons and retrotransposons, with the proportion in overall coverage remaining fairly stable throughout the phylogeny (Fig. 3c, Supplementary Fig. 2, Supplementary Data 5). Overall, the bulk of the expansion seems to have resulted from the activation of existing TE families, as several TE families present in the Antarctic clade are also present at low copy numbers in the Bovichtidae. Few TE families were observed exclusively within individual clades, although some unclassified TE elements remained in the dataset even after extensive manual curation.

In notothenioids, the capacity of transposons to generate evolutionary novelty and shape the evolutionary potential of whole lineages^44^ could be linked to the development of the adaptive features that characterise this radiation. To assess the influence of these transposition events on the genomic evolution of notothenioids, we selected the antifreeze glycoprotein (*afgp*) and the haemoglobin genomic loci as representative models for in-depth examinations.

### Evolution of the antifreeze glycoprotein gene family

The appearance of the antifreeze glycoprotein genes (*afgp)* in Antarctic notothenioid fishes was probably the most important innovation enabling survival in the sub-zero waters of the Southern Ocean. AFGPs prevent organismal freezing by binding to ice crystals that enter the body, thereby arresting ice growth^51,52^. The multigene *afgp* family encodes an array of AFGP size isoforms, whereby each gene is composed of two exons, exon 1 (E1) encoding a signal peptide, and exon 2 (E2) encoding an AFGP polyprotein^10,53^ (Supplementary Fig. 3a). The long polyprotein precursor comprises many AFGP molecules composed of varying numbers of repeats of a tripeptide (Thr-Ala-Ala), linked by conserved three-residue spacers (mostly Leu-X-Phe), which on post-translational removal yield the mature AFGPs^10,53,54^. Taken together, the tandemly arrayed *afgp* copies with their extremely repetitive coding sequences present formidable bioinformatic challenges, precluding accurate sequence assemblies and reconstructions of the entire antifreeze gene locus from genomic data until now. The most comprehensive representation of the locus to date was assembled from Sanger-based sequencing of BAC clones for *D. mawsoni*, although this still contains gaps and uncertainties^55^. Furthermore, some aspects of the evolutionary derivation of *afgp* from its *trypsinogen-like protease* (*tlp*) ancestral gene have not yet been fully resolved. Other uncertainties include why copies of the chimeric *afgp/tlp* genes, proposed to be evolutionary intermediates^53,56^, persist in extant genomes of the cryonotothenioids, the origin of the three-residue linker sequence in the AFGP polyprotein, and finally, the mechanism of expansion of *afgp* copies^55^.

**Fig. 4:**
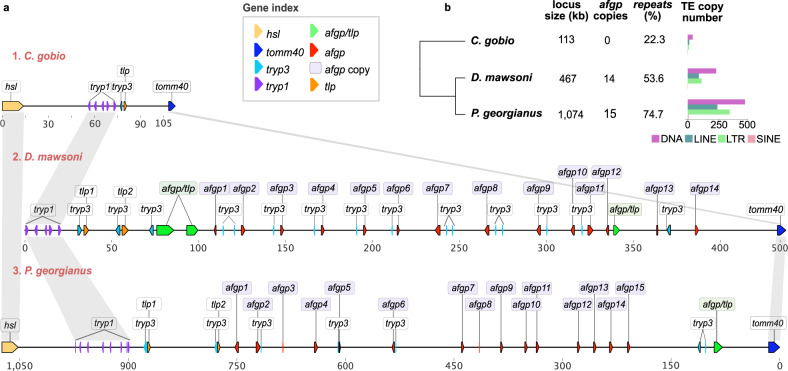
Reconstruction of the antifreeze locus. **a** Reconstructed physical map of the antifreeze locus for three notothenioid species: (1) *C. gobio*, which represents the ancestral state of the locus, (2) *D. mawsoni*, and (3) *P. georgianus*, which represent derived loci. The *C. gobio* and *D. mawsoni* loci are shown at the same scale, and the *P. georgianus* locus is shown in half scale and reverse orientation. Coloured triangles represent different genes and lilac rectangles represent *afgp* copies (see gene index). *afgp*: antifreeze glycoprotein genes, *tlp*: trypsinogen-like protease, *tryp1*: trypsinogen1, *tryp3*: trypsinogen3 (both *tryp1* and *tryp3* are *prss59* homologues), *tomm40*: translocase of outer mitochondrial membrane 40 homolog, *hsl*: hormone sensitive lipase (lipeb), afgp/tlp: chimeric afgp and tlp gene. **b** Cladogram of the three species analysed, with total length of locus, repeat content (%), number of *afgp* gene copies, and number of transposon copies annotated in each species locus (including DNA, LINE, LTRs, and SINE elements). Colours represent different TE classes as shown in legend. Source data are provided as a Source Data file.

Using long read data, we assembled the entire *afgp* locus into a single contiguous genomic sequence for a derived icefish species (*P. georgianus*). We also assembled the region corresponding to this locus in a species from a clade that separated prior to the appearance of *afgps* (*C. gobio*) (Fig. 4a.1,3). For comparison, we reanalysed the *afgp* locus of *D. mawsoni* (Fig. 4a.2), which represents one of the earlier diverging lineages of cryonotothenioids after *afgp* emergence^55^. In addition, we annotated *afgp* genes in three more genomes representing three different cryonotothenioid families (*H. antarcticus, T. bernacchii, G. acuticeps*) and located them in multiple scaffolds (Supplementary Fig. 4). Manual reassembly to resolve the breaks in these gene clusters in these three species was not possible due to the lack of sufficiently rich long-range data. The assembly of the *afgp* locus in *P. georgianus*, which spans more than 1 Mb in length (1074 kb), was manually curated to correct mis-assemblies and to verify gene completeness (Methods). The *P. georgianus* locus is approximately ten times the length of the corresponding region (113 kb) in *C. gobio*, and more than twice the length of the intermediate *D. mawsoni* locus (467 kb). Overall we observe a remarkable locus size expansion that appears to have accelerated in icefishes (Fig. 4a.3).

Annotation of the *afgp* genes could not be extended to the short read assemblies due to limitations of the length of the sequencing data and the complexity of the locus. The *afgp* genes typically contain >1 kb to ~7 kb of extremely repetitive sequence due to the nine-nucloetide repeats encoding the many AFGP molecules in the polyprotein (tripeptide Thr-Ala-Ala cds) (Supplementary Fig. 3b), and this precludes correct assembly using 150 bp Illumina data. Even if the overall repeat coverage of the region is estimated, this still does not allow for an accurate estimate of the gene copy number due to the length variability of individual genes^55^. We therefore focused on species with long read data for copy number estimation (Supplementary Fig. 4), and those with structurally complete loci for additional analysis (Fig. 4).

We identified 15 *afgp* genes in *P. georgianus* (Fig. 4a.3), of which eight are structurally intact and expected to be functional, while the other seven contain various mutations and are thus potentially pseudogenes. In addition, there is one chimeric *afgp/tlp* gene, previously regarded as a putative evolutionary intermediate of *afgp* genes. This also appears to be a pseudogene, because it lacks the signal peptide exon-1, possesses a premature termination codon in the AFGP-coding exon-2, and exon-2 would encode a single long run of 722 Thr-Ala-Ala tripeptide repeats (~6.5 kb) without any of the conserved, cleavable tripeptide linker sequences of the functional *afgp* polyprotein genes (Supplementary Fig. 3b**)**. Re-annotation of the *D. mawsoni afgp* region identified 14 *afgp* copies, and three chimeric *afgp/tlp* genes, one of which appears to be complete in terms of reading frame and cleavage sites, and therefore potentially functional (Fig. 4a.2).

The large size discrepancy between the *P. georgianus* and *D. mawsoni afgp* locus cannot be explained by the expansion of *afgp* genes alone, as only one extra copy was found in the icefish species, but instead seems to be primarily due to an expansion of TEs. The repeat content of the locus substantially exceeds the average TE content of the respective genomes (*D. mawsoni*: 53.6% compared to 40.1% genome average; *P. georgianus:* 74.6% compared to 54.3%), consistent with a bias towards TE insertion or retention. We further found evidence of multiple new TE insertions in the region that include representatives of seven LTR, six LINE, and 18 DNA TE families, as well as large expansions of Gypsy, L2, hAT-ac, and Kolobok-T2 copy numbers (Fig. 4b, Supplementary Table 1, Supplementary Fig. 5). Hence, in addition to transposon copy number being increased by segmental tandem duplication with the *afgp* genes, there has also been active transposition into this region, with the new TE copies potentially involved in local rearrangements.

The physical map of the *afgp* locus presented here for *P. georgianus* represents the most complete reconstruction to date for any icefish species (Fig. 4a.3). Previous attempts to map the locus using short read generated assemblies^31^ identified only four to eight copies of the antifreeze genes in various notothenioid genomes. A long-read assembly of *Chaenocephalus aceratus* (blackfin icefish) revealed 11 *afgps*^15^. The locus is also found to be highly fragmented in a recent chromosome-level genome assembly of *D. mawsoni*^57^, once more demonstrating the challenge of assembling this region. Our assemblies also include finer mapping of co-localised, and potentially co-evolving, gene families such as the trypsinogen genes, and tracking of the evolution of the chimeric intermediate gene (*afgp/tlp*). Finally, we observed an inversion of the locus in the icefish *P. georgianus* in comparison to the *D. mawsoni* haplotype 1 reconstruction^55^.

Two important points emerge regarding the evolutionary progression of the *afgp* gene locus. First, the presence of varying numbers of apparently functional as well as multiple pseudogene copies of *afgp* genes indicates that the locus remains evolutionarily dynamic. We suggest that the maintenance of functional copies in a protein gene family is driven by the strength of selective pressures exerted by the environment. *P. georgianus* is distributed in the considerably milder lower latitudes of the Southern Ocean, around the North of the West Antarctic Peninsula and the Scotia Arc Islands^58^ (Fig. 1b), but has never been found in the colder high-latitude waters. Thus, degeneration of previously functional copies would be consistent with a relaxation of selection on maintaining a large functional copy number and an energetically costly high level of protein production. Second, the chimeric *afgp/tlp* gene, which was earlier considered to be an evolutionary intermediate state of the early *afgp* genes, can still be found in the icefish (Fig. 4). Whilst one chimeric copy remains, it has sustained premature protein truncation and mutation (Supplementary Fig. 3B). This is in contrast to the presence of multiple chimeric genes and apparently at least one functional copy in *D. mawsoni*^55^. The chimeric gene in *P. georgianus* is clearly a pseudogene on its way to extinction. First, it lacks the signal peptide coding sequence, thus could not produce a secreted protein even if functional. Second, the very long run of coding sequence of *afgp* tripeptide repeats (722 repeats; ~6.5 kb) without any of the conserved cleavable 3-residue linker sequences in functional *afgp* polyprotein genes is indistinguishable from simple sequence repeats of nine nucleotides. This suggests that once independent functional *afgp* copies were formed and with independent *tlp* already present, the maintenance of a chimeric copy may have become unnecessary in the less selective lower latitude habitat ranges that *P. georgianus* colonised.

The evolutionarily dynamic nature of the notothenioid *afgp* gene family can also be gleaned from the sequence of *T. bernacchii*, which is a species that resides in the most severe conditions at the southernmost limit for marine life in the Southern Ocean (McMurdo Sound, 78^o^S). Even though its *afgp* locus assembly lacks contiguity, its annotation presents the largest set of *afgp* gene copies of all analysed species (24 copies, of which at least 11 are apparently functional), and also maintains three chimeric genes (Supplementary Fig. 4). Future efforts in assembling the challenging *afgp* loci to contiguity for cryonotothenioids across latitudinal clines will inform on the evolutionary dynamics of the adaptive *afgp* trait as driven by environmental selective strength.

### Gene gains and losses drive haemoglobin evolution in notothenioids

The haemoglobin gene family has also been under strong selective pressure in notothenioids^59^. Haemoglobin is essential for oxygen transport, and the evolution of haemoglobin genes has been fuelled by duplications that enabled diversification of paralogous genes, as well as adaptation to changing environments through alterations in expression patterns^60,61^. In teleosts, haemoglobins are organised in two clusters, each containing both α and β globin genes: the larger MN cluster (flanked by genes *kank2* and *nprl3*), and the smaller LA cluster (flanked by *rhbdf1b* and *aqp8*)^62^. Relaxed selection on haemoglobins and red blood cells in the cold, oxygen-rich Southern Ocean has led to moderate to severe anaemia in multiple notothenioid lineages^63^. The icefish family (Channichthyidae), often called “white blooded” icefishes due to their translucent white blood, are the only known vertebrates that completely lack haemoglobin and do not produce mature erythrocytes. Instead, they rely on oxygen physically dissolved in the blood plasma, which is possible because of the high oxygen saturation level at the freezing temperatures of the Southern Ocean^64^. The mechanisms underlying the loss of haemoglobin genes in the icefish are still a mystery, partly because the reconstruction of complete haemoglobin loci was not possible with previous fragmented genome assemblies. Here we describe the most complete reconstruction of the haemoglobin loci in notothenioids to date, allowing us to evaluate their evolution, track the loss of haemoglobins in the icefish, and identify the potential involvement of transposable elements in this process.

**Fig. 5:**
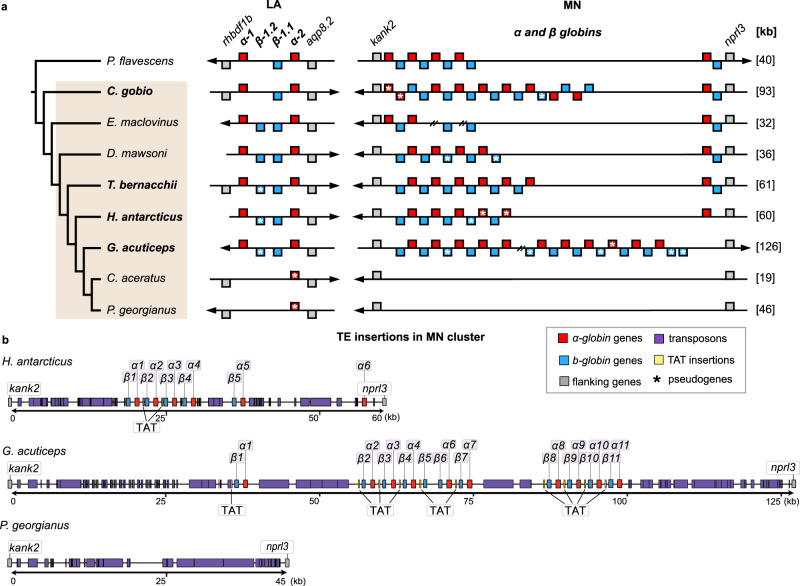
Reconstructed synteny of haemoglobin loci and TE insertions. **a** Species analysed and syntenic reconstruction of LA and MN haemoglobin gene clusters. **b** Transposon insertions in the MN region of *H. antarcticus*, *G. acuticeps*, and *P. georgianus* genomes. Red: α*-globin* genes, blue: *β-globin* genes, grey: flanking genes, purple: transposon insertions, yellow: TAT-like repeats. Pseudogenes are marked with asterisks. Breaks in the assembly are indicated with double forward slashes. Bold face indicates species sequenced in the present study. Arrows show locus orientation and total lengths of MN locus in different species are given in brackets (kb, at right). Source data are provided as a Source Data file.

Using five new long-read assemblies, we achieved the contiguous assembly of both LA and MN haemoglobin gene clusters for most of the species, including their flanking genes^62,65^, and compared these with four published assemblies for three notothenioids (*Eleginops maclovinus*^32^, *D. mawsoni*^57^, and *C. aceratus*^15^) and one temperate non-notothenioid perciform (*Perca flavescens*^66^) (Fig. 5a, Supplementary Fig. 6). The two loci were found to be distinct^67^ and located on two different chromosomes (LA: chr19, MN: chr8) that originated in the teleost genome duplication^68^. We devised and use here a new naming system for teleost haemoglobin genes independent of the life stage of expression (Methods). In the icefish species examined, we confirm the complete loss of functional haemoglobin genes in both clusters, with a single pseudogenized gene copy remaining in the LA locus. Synteny analysis suggests that the loss of haemoglobins potentially occurred as a single event for each locus. Furthermore, we find that the remaining length of the MN locus in *P. georgianus* is characterised by multiple transposon insertions (Fig. 5b). No other coding genes are found in the region, and the total length of the remaining genomic region is approximately the same size as that of red-blooded cryonotothenioids (30-60 kb). The most common transposon insertions include LINE/L2 elements, which account for ~20% of the total length of the *P. georgianus* MN locus (Supplementary Table 2, Supplementary Fig. 7). Similar transposon insertions are found in the LA locus, where the only haemoglobin remnant is the third exon of the pseudogenized *α-globin.2* (Fig. 5b), as previously identified^15,67,69^.

In contrast, in red-blooded species, haemoglobin loci are characterised by several local duplications (Fig. 5). For the LA cluster, in the common ancestor of *E. maclovinus* and cryonotothenioids we find a previously unidentified duplication of the *β-globin.1* gene that gave rise to two *β-globin* copies (*β-globin1.1* and *1.2*) (Fig. 5a). Subsequently, one duplicated copy was repeatedly lost or deleteriously altered in other cryonotothenioid species, while the other copy was retained throughout, until completely lost in the icefish. In the larger MN cluster, the number of haemoglobin gene copies varies considerably across species, with five to 11 *α-globin* and five to 13 *β*-globin genes, while containing multiple lineage-specific tandem duplications of the *α-globin.1* and *β*-*globin.1* gene pair in every lineage analysed (α-globin and β-globin genes, Fig. 5a). We retrieved a lower number of copies in the MN locus of the one species for which short read data were used for assembly (*E. maclovinus*^32^), potentially due to incomplete assembly or misassembly. Several cryonotothenioid species display lineage-specific frame-shifts or premature stop codons predicted to cause loss of function. The nature of the pseudogenisation of these gene copies suggests multiple independent pseudogenisation events. Only the loss of the first *α-globin.1* copy appears to be shared across cryonotothenioids. At the opposite end of the cluster, the *α-globin.2* and *β-globin.2* pair was retained with, however, the notable loss of *β-globin.2* in *H. antarcticus*, and in *G. acuticeps*. The case of *G. acuticeps* is particularly interesting, as here we identify a massive expansion of haemoglobin genes in the MN cluster, comprising more than twice as many haemoglobin genes as in other cryonotothenioid species. *G. acuticeps* is a member of the Bathydraconidae (dragonfishes), the group most closely related to the haemoglobin-lacking icefish. Furthermore, we find that each haemoglobin gene pair is preceded by a TAT-like repeat insertion, consistent with non-homologous recombination between these repeats underlying the tandem expansion of gene pairs (Fig. 5b). In other globin genes, such as myoglobins, TAT-like insertions have been shown to interfere with transcription regulation^70,71^ raising the possibility that these insertions as well as overall copy number are affecting expression levels. Further analysis of haemoglobin expression levels would be needed to understand how these TAT-like insertions influence haemoglobin transcription regulation in notothenioids.

By assembling the most complete reconstruction of notothenioid haemoglobin loci to date, we track the patterns of loss and gain of haemoglobin copies across the radiation and identify transposon insertions that may have influenced their evolution, while syntenic analysis suggests that the loss of haemoglobins in icefish could be potentially attributed to a single deletion event. Though it has been suggested that the lack of erythrocytes may reduce blood viscosity, the energy required to pump high volumes of blood and the need for additional physiological adaptations put into question whether the loss of haemoglobins in icefishes is indeed an adaptive trait^4,59^. Perhaps the lack of intense niche competition aided the establishment of this phenotype in the highly oxygenated waters of the Southern Ocean in the past. However, relying on an oxygen absorption system that depends on oxygen diffusion leaves the icefish intensely vulnerable to rising temperatures and thus decreased dissolved oxygen in the future^64^.

In conclusion, we present the most extensive effort to date to investigate the genomic evolution underlying the iconic notothenioid fish radiation, via the generation of a set of 24 new genome assemblies encompassing representatives from almost all notothenioid families. We demonstrate that the use of high-quality genome assemblies over a wide taxonomic breadth can help to decipher the evolutionary history of this recent vertebrate radiation. We identify critical steps in the evolution of key gene families that involve large genomic rearrangements in repetitive regions that could only be reconstructed with the aid of long-read assemblies. We show that the evolutionary history of the remarkable notothenioid radiation was associated with, and potentially driven by, transposon proliferation, which could have affected the evolutionary advantage of these species during freezing events occurring in the Southern Ocean. In particular, TE expansion events can be linked to the structure of characteristic repetitive gene families such as the haemoglobin and antifreeze genes. Beyond these direct insights, our work provides an extensive resource for the future study of notothenioid genomic evolution, enabling further research to advance our understanding of the notothenioid radiation, and of genomic adaptations to extreme environments more widely. As the fate of notothenioid diversity is linked to very narrow margins of temperature tolerance, studying their adaptability is particularly relevant now with the unfolding climate crisis and the warming of the Southern Ocean^72^.

## Methods

### Sample processing and sequencing

Tissue samples were collected in compliance with all relevant ethical regulations and either frozen immediately at −80 ˚C, or preserved in ethanol and then frozen to preserve the quality of genomic DNA. Tissue preservation can influence the quality of extracted DNA^73^, and flash freezing is the optimal preservation method for use with long-read sequencing. High molecular weight DNA (HMW DNA) was extracted using the Bionano Agarose plug extraction protocol^74^ or a modified version of the MagAttract kit (Qiagen) (Supplementary Data 1). The quantity of extracted HMW DNA was evaluated with the HS Qubit DNA kit and the fragment profile and overall quality was assessed with the Femto Pulse instrument (Agilent). Pacific Biosciences (PacBio) sequencing was performed with CLR (Continuous Long Reads) SMRT cells. PacBio libraries were made using the SMRTbell Template Prep Kit 1.0, following the PacBio protocol. A size selection was performed on the BluePippin instrument (Sage Science), with a 15 kb cut off and sequence data were generated on the Sequel instrument using Seq kit v2/Binding Kit v2.0 with a 10 h movie. Illumina sequencing paired-end (PE) libraries were generated for eight species and sequenced by multiplexing two species per lane on Illumina HiSeqX (150 bp PE). Linked reads 10X Genomics Chromium sequencing was performed for 17 species (Table 1). Linked-read libraries were prepared using the Chromium Genome Reagent Kit, and the Chromium Genome Library Kit & Gel Bead Kit, according to the manufacturer’s instructions^75,76^, with standard DNA input (1 ng). Hi-C libraries were generated with a Dovetail kit for *P. georgianus* and with ARIMA Genomics kit for *C. gobio*, and each was sequenced on the Illumina HiSeq4000 platform. Bionano Irys optical mapping was used for scaffolding the assembly of *H. antarcticus*, as well as that of *C. gobio*^23^.

Total RNA for RNAseq was extracted using the RNeasy extraction kit (Qiagen), from ~20–40 mg of tissue. The RNA quality was assessed with the Qubit HS RNA kit and Agilent Bioanalyzer Nano chips, and only extracts with RIN value > 8 were used for sequencing. Illumina 150 bp PE libraries were prepeared and sequenced on the HiSeq4000 platform. For *C. gobio* four tissues were used including brain, muscle, and ovary from one individual, preserved in RNAlater, and frozen spleen from a different individual. For *T. bernacchii* four RNAlater preserved tissue types were used including brain, muscle, and ovary, and testis from a second individual, For *G. acuticeps* two tissues were used, including brain and ovary from one individual, preserved in RNAlater. All sample processing and sequencing was performed at the Wellcome Sanger Institute, UK.

### Genome assembly and curation

For genome assembly we used a combination of different sequencing technologies, which were either used in conjunction (hybrid assemblies) or individually. Our genome assemblies were generated as follows.

For *C. gobio* the assembly was generated based on 75x PacBio Sequel data, 54x Illumina HiSeqX data generated from a 10X Genomics Chromium library, Bionano Saphyr two-enzyme data (Irys) and 145x coverage HiSeqX data from a Hi-C library (for Hi-C, tissue from a different individual was used), as described in ref. ^23^. An initial PacBio assembly was generated with Falcon-unzip without repeat-masking during overlap detection. The primary contigs were first scaffolded using a wtdbg^77^ assembly as a guide, then scaffolded further using the 10X data with scaff10x and then with Bionano two-enzyme hybrid scaffolding. After using the PacBio data to gap-fill with PBJelly and polish with Arrow, the assembly was polished again using the 10X Illumina data and freebayes. Contiguity was then further increased by filling gaps with the contigs from a wtdgb assembly made from Canu^78^ corrected PacBio reads. The assembly was then re-polished with Arrow and freebayes, and retained haplotigs were identified with purge_haplotigs^79^ and scaffolded to chromosomes using Arima Hi-C.

For *P. georgianus* the assembly was based on 93x PacBio, 56x 10X Genomics Chromium, and Dovetail Hi-C data. An initial assembly was generated using Falcon-unzip, retained haplotig identification with purge_haplotigs, with 10X based scaffolding with scaff10x, BioNano hybrid-scaffolding, Hi-C based scaffolding with SALSA2^18^, Arrow polishing, and two rounds of FreeBayes^80^ polishing. This assembly comprises 24 chromosomes, which were numbered in correspondence to the medaka HdR1 assembly (*Oryzias latipes*, GCA_002234675.1), as for the *C. gobio* genome^23^.

The assembly for *H. antarcticus* was based on 67x PacBio data, 40x of 10X Genomics Chromium data, and Bionano Irys data. An initial PacBio assembly was generated with Falcon-unzip, retained haplotig identification with purge_haplotigs^79^, 10X based scaffolding with scaff10x, Bionano hybrid-scaffolding, Arrow polishing, and two rounds of FreeBayes polishing. For *T. bernacchii* and *G. acuticeps* the assemblies were based on PacBio and 10X data. For *T. bernacchii*, we used 46x PacBio data and 53x of 10X Genomics Chromium data, while the assembly for *G. acuticeps* was based on 31x PacBio data and 41.8x of 10X Genomics Chromium data. These were assembled using Falcon-unzip, 10X based scaffolding with scaff10x, Arrow polishing, and two rounds of FreeBayes polishing. Purge_dups^81^ was run on the curated assemblies to further remove retained duplications.

Finally, to improve the quality of the PacBio assemblies we performed manual curation to remove mis-assemblies, duplications, and sequencing contamination, and to merge scaffolds based on supporting evidence, which has been shown to substantially improve the continuity and accuracy of genome assemblies^20^. Each assembly was manually curated using the Genome Evaluation Browser (gEVAL)^19^. Scaffold integrity was confirmed with PacBio read mapping and enhanced with 10X illumina read mapping, read information and contig end sequence overlaps. While for *H. antarcticus* scaffold integrity was further confirmed using Bionano BssSI optical maps, visualised in Bionano Access, breaking and re-joining where necessary. For *P. georgianus* a 2D map was built using Hi-C reads, allowing further scaffold correction and super-scaffolding to bring the assembly to chromosome scale. Artificially retained haplotypic duplications were removed with purge_dups^81^ (Supplementary Table 3).

For 19 more species only 10X Chromium or Illumina HiSeqX were used for assembly (Table 1). For 11 species sequenced with 10X Genomics Chromium data, genome assembly was performed using Supernova 2.0 (Supernova 2.0 Software). After initial assembly, retained haplotigs were identified using purge_haplotigs^79^. For the remaining eight species which were only sequenced with Illumina HiSeqX, a primary assembly was generated with a reference guided approach using SOAPdenovo2^25^. The short insert reads were initially base error corrected using BFC (https://github.com/lh3/bfc). After this step, larger kmer sizes (e.g. 70), may be applied to improve assembly. SOAPdenovo^25^ was used to process the cleaned short insert reads, followed by GapCloser for contig gap filling. The scaffolds were further enhanced by the use of cross_genome, a tool which maps genome synteny to merge scaffolds (Phusion2 - Browse /cross_genome), as has been previously applied in genome assemblies such as Tasmanian devil^82^ and grass carp^83^. To finalise these assemblies, decontamination methods were used to remove contaminants from sequencing (e.g., adapter sequences) or symbionts. Metrics for sequencing data and assemblies are provided in Supplementary Data 3 and 6, and Supplementary Table 4.

### Gene annotation

Gene annotation was generated for all five PacBio assemblies, using the Ensembl Gene Annotation system as follows^27^. Annotation was created primarily through alignment of short read RNAseq data to the genome. Gaps in the annotation were filled via protein-to-genome alignments of a select set of vertebrate proteins from UniProt^84^, which had experimental evidence for existence at the protein or transcript level.

At each locus, the data were collapsed and consolidated, with priority given to models derived from the RNAseq data, producing a set of final gene models along with their associated non-redundant transcript set. To help differentiate between true isoforms and fragments, the likelihood of each Open Reading Frame (ORF) was assessed in relation to known vertebrate proteins. Low-quality transcript models, e.g. those with evidence of a fragmented ORF, were removed. In loci where the RNAseq data were fragmented or missing, homology data took precedence, with preference given to longer transcripts that had strong intron support from the short-read data.

Gene models from the above process were classified into three main types: protein-coding, pseudogene, and long non-coding. Models with hits to known proteins, and few structural abnormalities were classified as protein-coding. Models with hits to known proteins that also display abnormalities such as the absence of a start codon, non-canonical splicing, unusually small intron structures (<75 bp) or excessive repeat coverage, were reclassified as pseudogenes. Single-exon models with a corresponding multi-exon copy elsewhere in the genome were classified as processed (retrotransposed) pseudogenes. If a model failed to meet the criteria of any of the previously described categories, did not overlap with a protein-coding gene, and had been constructed from transcriptomic data, then it was considered as a potential lncRNA. Potential lncRNAs were additionally filtered to remove single-exon loci due to the unreliability of such models.

Putative miRNAs were predicted via a BLAST of miRBase^85^ against the genome, before passing the results to RNAfold^86^. Other small non-coding loci were identified by scanning Rfam^87^ against the genome (described in more detail in ref. ^27^) and passing the results into Infernal^88^.

Gene annotations are available on the Ensembl server under GCA_900634415.1 (database version 9.31^81^) for *C. gobio*, and on Ensembl Rapid Release (https://rapid.ensembl.org/) for *G. acuticeps*, *P. georgianus*, *H. antarcticus*, and *T. bernacchii* (Supplementary Data 4). Comparison of orthologous clusters was performed with OrthoVenn2^89^. For antifreeze and haemoglobin genes further manual curation was undertaken as described below.

### Transposable element annotation and analysis

De novo annotation of transposable elements was performed using RepeatModeler v.2.0^90^ and RepeatMasker v.4.0.1^91^. For each of the five PacBio assemblies (*C. gobio, T. bernacchii, H. antarcticus, G. acuticeps*, and *P. georgianus*) a de novo repeat library was generated using RepeatModeler2^90^. To enhance the detection of LTR retrotransposons, the programs LTRharvest^92^ and LTR_retriever^93^ were run as part of the RepeatModeler2 pipeline. To improve the quality of the annotation, the identified elements from each genome were further curated manually using the “BLAST, extend, extract” process^94^ to remove false assignments and achieve complete length elements. The consensus TE sequences that were identified by RepeatModeler2 were blasted against the genomes (BLAST+^95^), and the sequences of the 50 best hits were extracted along with a 1-kb long flanking sequence on each side. Multiple sequence alignments were generated for each set of top hits, using MUSCLE^96^. Each multiple sequence alignment was visualised with belvu^97^ and then manually inspected to confirm TE element completeness. TEs that appeared to be extending beyond alignment boundaries were subjected to additional rounds of curation, until the complete sequence was recovered. Finally, new consensus sequences were extracted from the multiple alignments using hmmer (http://hmmer.org/). Overall a total of ~2000 elements generated by RepeatModeler2 were manually curated. A custom TE library was created combining all the curated element outputs, and all genomes were masked with RepeatMasker (with options -rmblast -s).

To analyse the repeat content of each genome we used a Perl script to parse the RepeatMasker.out and.align files^98^. This was used to calculate the total amount of DNA of the genome and different categories of TEs (e.g. class, family), and the % of divergence from the consensus (Kimura divergence). The amount of DNA was then split in bins and plotted against the coverage to generate repeat landscape plots, which were made using ggplot2^99^ in R. The % divergence indicates the age of TEs, with lower percentage divergence from the consensus sequence indicating younger TEs (Fig. 3a). To examine the effect of TE expansions in genome size variation we correlated the total amount of DNA in TEs vs. assembly size using a linear regression model (Pearson correlation coefficient) (Fig. 3b). To plot annotated TEs colocalized with gene copies (Fig. 5b) we used the DNA Features Viewer library in python. Finally, TE copy numbers were calculated using another Perl script parsing the RepeatMasker output^100^ (Fig. 4b).

### Phylogenetic analysis

Phylogenetic analysis was performed using single copy ortholog genes identified with BUSCO^26^, for the 24 newly sequenced notothenioid genomes and 17 previously published genomes of seven notothenioids and ten further species of percomorph fishes. The species and assembly versions used are listed in Supplementary Table 5. BUSCO (v2) was run with lineage “actinopterygii_odb9”, and the sequences of single copy orthologs identified in each assembly and extracted for use in further analysis.

We used MAFFT v.7.453^101^ to align 266 selected BUSCO genes that were single copy in our annotated gene sets. The 266 alignments were inspected by eye, and apparently misaligned sequence regions were set to missing data. A total of 1,141,524 amino acids were set to missing out of 6,410,688, including nine alignments that were excluded completely, leaving 257 alignments for further analysis. We then aligned nucleotide sequences of the same BUSCO genes according to the amino-acid alignments, ensuring that regions corresponding to the removed sequences were again set to missing data in the nucleotide sequence alignments. Sites with high entropy (entropy-like score > 0.5) or high proportion of missing data (gap rate >0.2) were removed with BMGE v.1.1^102^ and alignments with more than three completely missing sequences, a minimum length below 500 bp, or a standard deviation of among-sequence GC-content variation >0.03 were excluded. These filters were passed by 228 alignments. For each alignment we performed gene-tree analyses using BEAST2 v.2.6.0^39^ with a Markov-chain Monte Carlo chain length of 25 million iterations, assuming the Yule model of diversification^103^ and the uncorrelated lognormal relaxed clock model^104^, and averaging over substitution models with the bModelTest add-on package^105^. These gene trees were time-calibrated by arbitrarily constraining their root age to 100 million years (with a standard deviation of 0.1). Chain convergence was suggested by effective sample sizes (ESS) per parameter >200.

We identified the most suitable alignments for further phylogenomic analyses based on the minimum ESS value per alignment and estimates for the mutation rate and its among-species variation. We compiled a “strict” set of alignments that included all those that had a mean mutation rate estimate below 0.002 per bp per million year, a mutation rate standard deviation (relative to the mean estimate) below 0.9, and a minimum ESS value >100; this set was a subset of a second, “permissive” set of alignments in which we placed those that had a mean mutation rate estimate below 0.00025 per bp per million years, a mutation rate standard deviation below 1.1, and a minimum ESS value >50. The strict and permissive sets contained 140 and 200 alignments, respectively.

For the strict set of 140 alignments, the permissive set of 200 alignments, and the “full” set of 257 alignments, we performed maximum-likelihood phylogenetic analyses with IQ-TREE v.1.7^41^ after alignment concatenation, maintaining separate partitions with unlinked instances of the GTR+Gamma substitution model for each of the original alignments. Node support was assessed with 1000 ultrafast bootstrap replicates^106^. Each of the three analyses was complemented with an estimation of gene- and site-specific concordance factors, and the three resulting sets of gene trees were used for separate species-tree analyses with ASTRAL v.5.7.3^42^.

Finally, we estimated the phylogeny and the divergence times of notothenioid species with BEAST2 from a concatenated alignment combining all alignments of the strict set. To avoid potentially saturated sites, we excluded all third codon positions from this analysis, and to reduce its computational demand we grouped 280 original data blocks (separating first and second codon positions for each of the 140 original alignments of the strict set) into 12 partitions selected with the cluster algorithm of PartitionFinder v.2.1.1^107^, assuming linked branch lengths, equal weights for all model parameters, a minimum partition size of 5000 bp, and the GTR+Gamma substitution model. The same substitution model was also assumed in the BEAST2 analysis, together with the birth-death model of diversification^108^ and the uncorrelated lognormal relaxed clock model^104^. Time calibration of the phylogeny was based on four age constraints defined according to a recent timeline of teleost evolution inferred from genome and fossil information^33^, at the most recent common ancestors of clades: Eupercaria, around 97.47 MYA (2.5–97.5 inter-percentile range: 91.3–104.0 MYA); the clade combining Eupercaria, Ovalentaria, and Anabantaria—around 101.79 MYA (95.4–109.0 MYA); the clade combining these four groups with Syngnatharia and Pelagiaria—around 104.48 MYA (97.3–112.0 MYA); and the clade combining those six groups with Gobiaria—around 107.08 MYA (100.0–114.0 MYA). All constraints were implemented as lognormal prior distributions with mean values as specified above and a standard deviation between 0.033 and 0.036. In addition, we constrained the unambiguous^33,109–111^ monophyly of the groups Notothenioidei, Perciformes, Ovalentaria, Anabantaria, and the clade combining the latter two groups. We performed six replicate BEAST2 analyses with 330 million MCMC iterations, and convergence among MCMC chains was confirmed by ESS values >120 for all model parameters and >270 for the likelihood and the prior and posterior probabilities. The posterior tree distribution was summarised in the form of a maximum-clade credibility tree with TreeAnnotator v.2.6.0^112^. We attempted to repeat the BEAST2 analyses with the permissive and full datasets, but these proved too computationally demanding to complete, so that even after 330 million MCMC iterations and run times of several months, some of the ESS values remained below 100. Nevertheless, the preliminary results from these analyses supported the same tree topology as the analyses with the strict dataset.

To place the time calibrated phylogeny in the context of historic ocean temperature variation, we used estimated benthic oxygen values previously published in ref. ^36^. The moving average was plotted with geom_smooth in R using a generalised additive model (GAM: (y ~ s(x, bs = “cs”)).

### *afgp/tlp* locus annotation and reconstruction

The location of the *afgp* locus in each genome assembly was initially identified with BLAST+^95^ searches, using as queries a copy of *afgp* and other gene sequences annotated in the previously published *D. mawsoni* locus (accession HQ447059.1, haplotype1)^55^. Specifically, within the locus the following genes were used as queries: Antifreeze glycoprotein H1-A2 (*afgp*), Trypsinogen H1-1d (*tryp1*), Trypsinogen H1-3a (*tryp3*), Trypsinogen-like protease 1 (*tlp*), Translocase of outer mitochondrial membrane 40 (*tomm40*), hormone sensitive lipase HSL (*lipeb*; for this gene a full length transcript was obtained independently and used as query)^55^. The exact location of each gene copy was confirmed and annotated manually, to identify numbers and sizes of exons. Each *afgp* copy was manually inspected for the presence of frame shifts and gaps to identify complete genes and pseudogenized copies.

All five genomes sequenced with PacBio contained copies of all six genes used for BLAST+ analyses (*afgps* and flanking genes) (Fig. 4, Supplementary Fig. 4). To further improve the assembly of the *afgp* locus on the *P. georgianus* genome, we mapped Falcon-corrected PacBio reads to the diploid assembly using minimap2^113^, and then filtered the mapped reads to remove secondary alignments (samtools view -F 256). We used GAP5^114^ to inspect and manually curate the mapped reads. Reads that mapped to more than one location were linked in GAP5, and by further inspecting these links and extending soft-clipped sequence, it was possible to merge contigs, resulting in a complete representation of the whole *afgp* gene locus. Finally, the reassembled sequence was polished using Racon^115^.

### New naming system for teleost haemoglobin genes

The current haemoglobin gene naming system in fish mostly relies on the zebrafish laboratory model species and on the expression pattern of each of its haemoglobin genes during embryonic and/or adult phases. While informative for zebrafish research, using a naming system based on embryonic or adult expression for species in which expression dynamics of haemoglobin genes cannot be assessed may lead to misinterpretations, especially because expression patterns of haemoglobin genes are known to be influenced by local organisation of the genomic region that may not be conserved across species^65,116^. Therefore, designating orthologous haemoglobin genes across species needs a nomenclature system that is independent of an expression pattern that may not be evolutionarily conserved. We thus propose here a novel naming system based on genomic organisation rather than expression data.

First, the established haemoglobin alpha and beta denominations (i.e., *hba* and *hbb*) are conserved due to clear sequence conservation. Second, the presence of each gene in the LA or the MN cluster is added as a suffix (e.g., *hbala* and *hbamn*). Third, a final numeral suffix is added to reflect the relative positioning of the gene within each cluster. The orientation of the most upstream *hba* gene determines the orientation of the cluster and is arbitrarily named *hbala1* and *hbamn1* for the LA and MN clusters, respectively. Thus, the nomenclature reflects position but not necessarily orthology. The neighbouring *hbb* gene is called *hbbla1* and *hbbmn1* for the LA and MN clusters, respectively. The names of genes further to the conventional right of the locus are suffixed with incremental numbers following the orientation of the cluster. Tandem duplicated genes (e.g., *hbamn1.1* and *hbamn1.2*) and pseudogene (e.g., *hbamn1.3p*) nomenclatures follow the Zebrafish nomenclature guidelines established by the Zebrafish Information Network (ZFIN)^117^.

### Haemoglobin gene locus annotation and reconstruction

To study haemoglobin genes in notothenioid species we focused on the five species (*C. gobio, T. bernacchii, H. antarcticus, G. acuticeps*, and *P. georgianus*) assembled with PacBio data, along with three previously published assemblies (*D. mawsoni*^57^*, E. maclovinus*^32^*,* and *C. aceratus*^15^) to provide as good a clade coverage as possible. The reference genome assembly of the yellow perch *(Perca flavescens*)^66^, a close relative to notothenioids within the order Perciformess^9^, was used as a reference to determine haemoglobin gene exon boundaries. We used flanking genes to confirm orthology between genes and clusters across species, and each exon of each gene was retrieved and their exact positions in the corresponding genome assembly was recorded. Using the *T. bernacchii* assembly as reference species we performed mVISTA^118^ alignments of genomic regions with LAGAN^119^ or Shuffle-LAGAN^120^. Protein sequences were aligned with MUSCLE^96^ and phylogenetic trees were reconstructed with RAxML-NG^121^ using the best-fitting substitution model according to ModelFinder based on Bayesian information criterion (BIC)^122^, 50 parsimony and 50 random starting trees, and 200 bootstraps or bootstopping at a default cut-off of 0.03 (protein alignments in Supplementary Data 7).

We used self-alignments with dotter^123^ to visualise the complete reconstruction of each haemoglobin cluster and we manually inspected each locus to identify possible gaps or mis-assemblies. We can confirm complete gapless assembly of the MN haemoglobin locus for each PacBio species, with the exception of one gap identified in the *G. acuticeps* genome, which could not be corrected with the available data (Fig. 5). Furthermore, the *P. georgianus* chromosomal assembly MN haemoglobin locus was also manually curated using GAP5^114^, as described above for the *afgp* locus.

Detailed information on all the tools and versions used for each analysis are provided in Supplementary Table 6.

### Reporting summary

Further information on research design is available in the Nature Portfolio Reporting Summary linked to this article.

## Supplementary information

Supplementary Information

Peer Review File

Reporting Summary

Description of Additional Supplementary Files

Supplementary Data 1

Supplementary Data 2

Supplementary Data 3

Supplementary Data 4

Supplementary Data 5

Supplementary Data 6

Supplementary Data 7

## Data Availability

The genome assemblies generated in this study have been deposited on NCBI under BioProject PRJEB53202 and the following accessions: *C. gobio*: GCA_900634415.1 (alt. hap. GCA_900634435.1), *T. bernacchii* GCA_902827165.1 (alt. hap. GCA_902827105.1), *H. antarcticus* GCA_902827135.1 (alt. hap. GCA_902827095.1), *G. acuticeps* GCA_902827175.1 (alt. hap. GCA_902827185.1), *P. georgianus* GCF_902827115.1 and GCA_902827115.2 (alt. hap GCA_902827155.1), *B. diacanthus* GCA_943590825.1, *B. variegatus* GCA_943593645.1, *T. loennbergii* GCA_943590855.1, *L. larseni* GCA_943594155.1, *L. squamifrons* GCA_943593335.1, *T. hansoni* GCA_943593355.1, *T. scotti* GCA_943590805.1, *L. nudifrons* GCA_943590975.1, *G. gibberifrons* GCA_943591055.1, *N. rossii* GCA_943590865.1, *D. longedorsalis* GCA_943591025.1, *H. velifer* GCA_943590885.1, *A. nudiceps* GCA_943590845.1, *B. marri* GCA_943591095.1, *V. infuscipinnis* GCA_943590875.1, *C. wilsoni* GCA_943593825.1, *C. antarcticus* GCA_943590835.1, *P. macropterus* GCA_943590895.1, *C. dewitti* GCA_943594065.1. Gene annotation for species *C. gobio* is available on Ensembl [www.ensembl.org], and for *T. bernacchii, H. antarcticus, G. acuticeps, P. georgianus* gene annotations are available on Ensembl Rapid Release [https://rapid.ensembl.org/]. RefSeq annotations for *C. gobio*, *T. bernacchii, G. acuticeps*, and *P. georgianus* can be found on NCBI under assembly accession numbers. All raw sequencing data are available on NCBI (accessions listed in Supplementary Data 1). Source data are provided as source data file. Data used for phylogenetic analysis along with alignments and phylogenetic trees are available on Dryad: 10.5061/dryad.80gb5mktn. Source data are provided with this paper.

## Source data

Source data
