# Supplementary material for: Genomics of cold adaptations in the Antarctic notothenioid fish radiation
Source: Nat Commun. 2023 Jun 9;14:3412. doi: 10.1038/s41467-023-38567-6 (PMC10256766; doi:10.1038/s41467-023-38567-6)
Supplement: Supplementary file 1 — Supplementary Information [file 41467_2023_38567_MOESM1_ESM.pdf]

Genomics of cold adaptations in the Antarctic notothenioid fish radiation

AUTHORS

Iliana Bista*[1,2,3,4], Jonathan M. D. Wood[1], Thomas Desvignes[5], Shane A. McCarthy[1,2], Michael Matschiner[6,7], Zemin Ning[1], Alan Tracey[1], James Torrance[1], Ying Sims[1], William Chow[1], Michelle Smith[1], Karen Oliver[1], Leanne Haggerty[8], Walter Salzburger[9], John H. Postlethwait[5], Kerstin Howe[1], Melody S. Clark[10], H. William Detrich III[11], C.-H. Christina Cheng[12], Eric A. Miska[1,3], Richard Durbin*[1,2]

AFILIATIONS

1 - Wellcome Sanger Institute, Tree of Life, Wellcome Genome Campus, Hinxton, CB10 1SA, United Kingdom
2 - Department of Genetics, University of Cambridge, Downing Street, Cambridge, CB2 3EH, United Kingdom
3 - Wellcome/CRUK Gurdon Institute, University of Cambridge, Tennis Court Rd, Cambridge, CB2 1QN, United Kingdom
4 - Naturalis Biodiversity Center, Leiden, 2333 CR, the Netherlands
5 - University of Oregon, Institute of Neuroscience, 1254 University of Oregon, 13th Avenue, Eugene OR 97403, USA
6 - University of Oslo, Natural History Museum, University of Oslo, Sars' gate 1, 0562 Oslo, Norway
7 - University of Zurich, Department of Palaeontology and Museum, University of Zurich, Karl-Schmid-Strasse 4, 8006 Zurich, Switzerland
8 - European Molecular Biology Laboratory, European Bioinformatics Institute, Wellcome Genome Campus, Hinxton, CB10 1SA, United Kingdom
9 - University of Basel, Zoological Institute, Department of Environmental Sciences, Vesalgasse 1, 4051 Basel, Switzerland
10 - British Antarctic Survey, High Cross, Madingley Road, Cambridge, CB3 0ET, United Kingdom
11 - Northeastern University, Department of Marine and Environmental Sciences, Marine Science Centre, 430 Nahant Rd., Nahant, MA 01908, USA
12 - Department of Evolution, Ecology, and Behaviour, University of Illinois, Urbana-Champaign, IL 61801, USA

***Corresponding authors:** Iliana Bista (ilianabista@gmail.com); Richard Durbin (rd109@cam.ac.uk)

Supplementary Information:

1. **Supplementary Figures**
2. **Supplementary Tables**
3. **Supplementary References**

1. SUPPLEMENTARY FIGURES

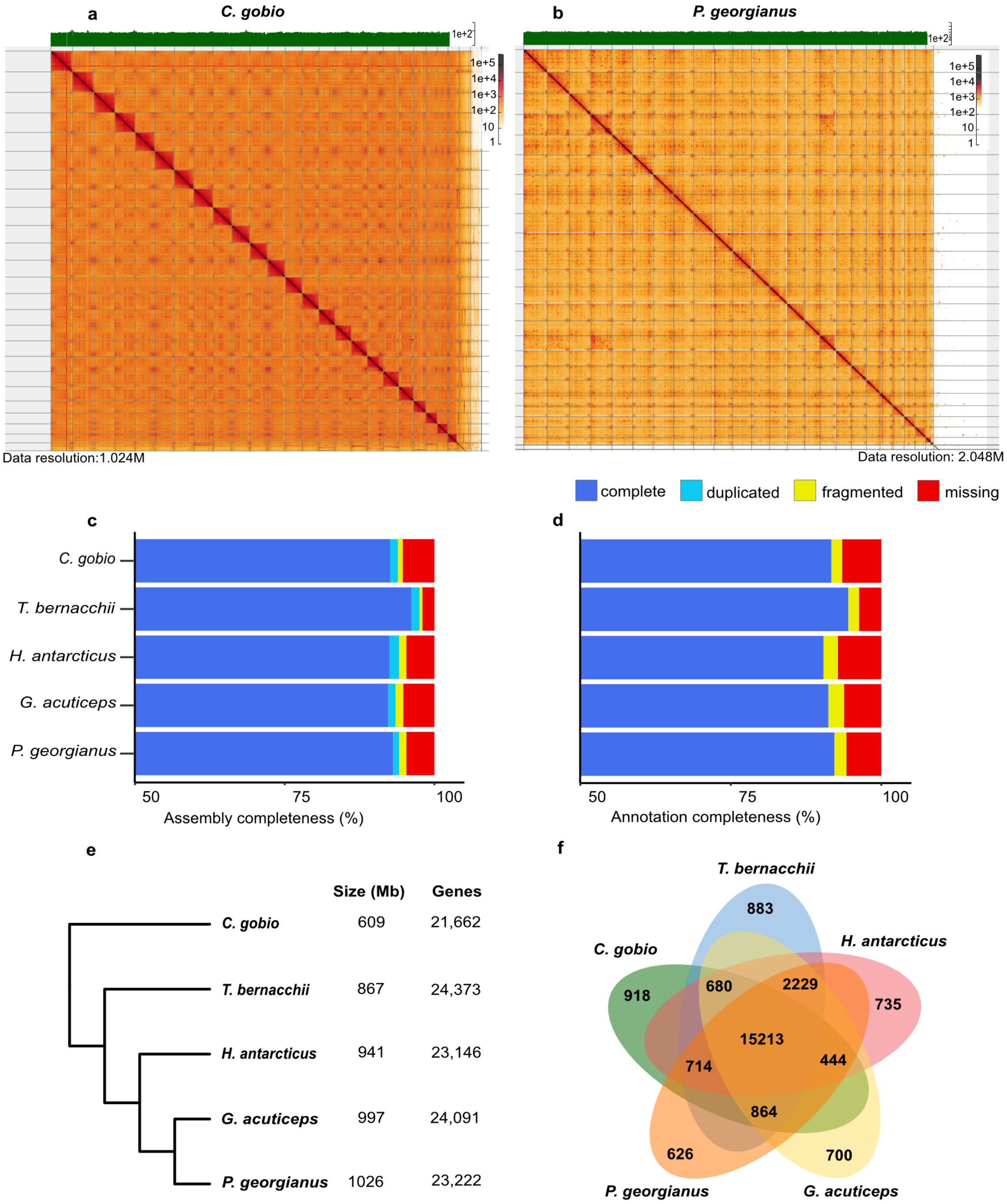

Supplementary Figure 1 | Hi-C maps and completeness scores for assemblies and annotations of the PacBio genomes. Hi-C maps for species *C. gobio* (a), and *P. georgianus* (b) generated with HighGlass, showing the assemblies scaffolded on 24 chromosomes (red squares across the diagonal), and read coverage (green track on top). c) Assembly and d) annotation completeness scores based on BUSCO (v.5), e) genome size and number of annotated genes (Ensembl) for each PacBio assembly, f) unique and shared Ensembl genes. Source data are provided as Source Data file.

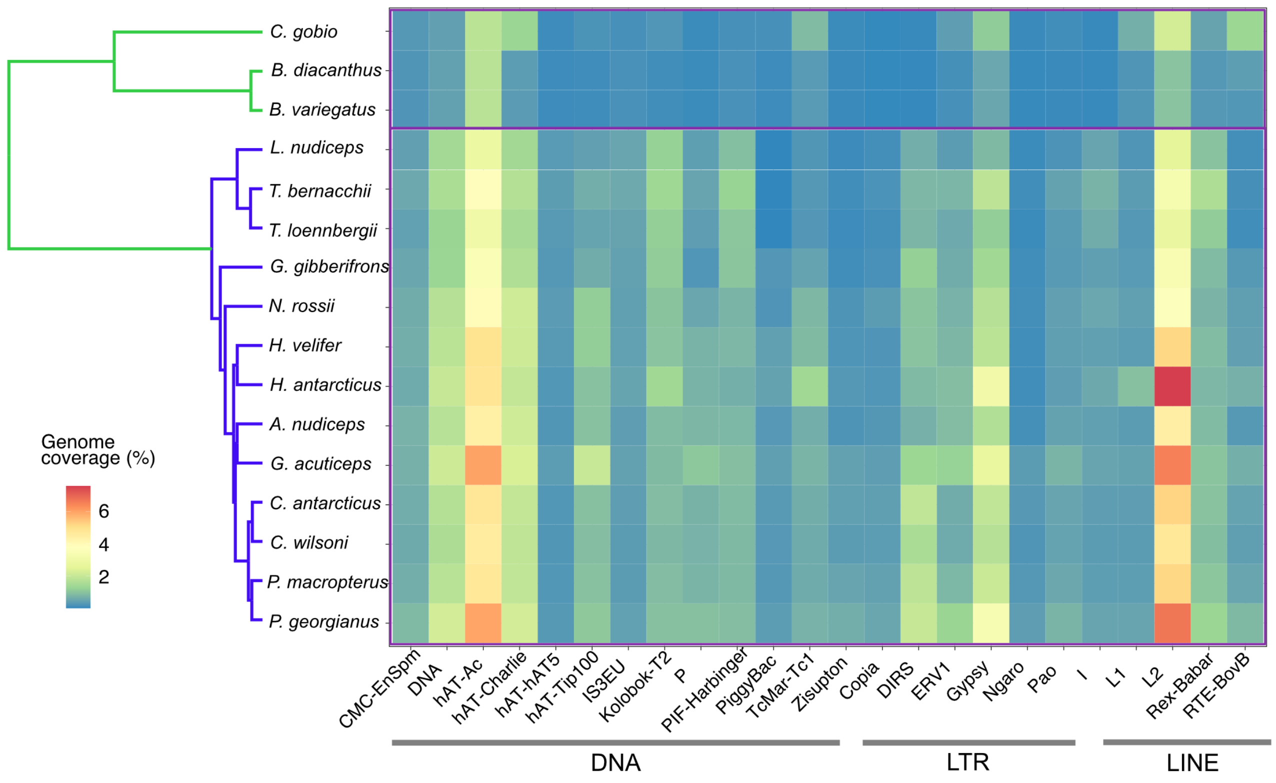

Supplementary Figure 2 | Heatmap of transposon coverage of most abundant transposon superfamilies. Coverage is shown as percent (%) of genome for 16 notothenioid species sequenced in present study, including DNA transposons, and LTR and LINE retrotransposons. The colour of tree branches indicates non-Antarctic (green), and cryonotothenioid (blue) species. Source data are provided as Source Data file.

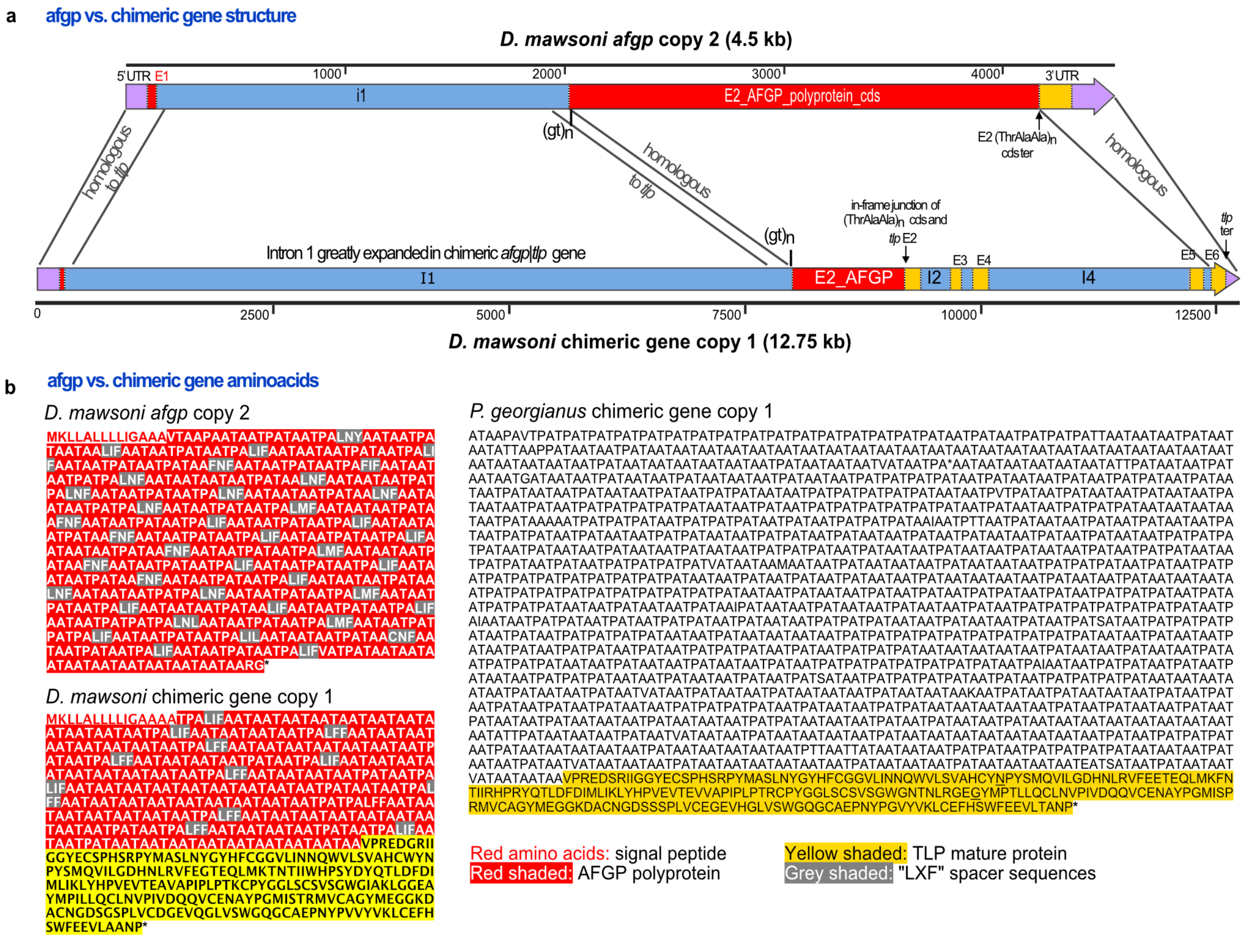

Supplementary Figure 3 | a) Structure of *afgp* gene copy 2 from species *D. mawsoni* showing the two exons (E1: signal peptide, E2: long polyprotein precursor), compared to a chimeric gene (copy 1 of *D. mawsoni*), which contains complete cds of both *afgp* and *tlp* genes. b) Amino acid sequences of *D. mawsoni afgp* copy 2 and chimeric gene copy 1, showing the signal peptide (red letters), AFGP polyprotein chain (red shading), the TLP mature protein chain (yellow shading), and spacer sequences (grey shading). Also shown is the remaining and pseudogenized chimeric gene in *P. georgianus*, which contains neither a signal peptide nor an AFGP polyprotein chain.

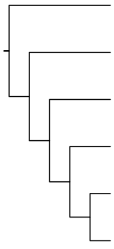

Genes and number of copies

	hsl	*tryp1*	*tryp3a*	*tlp*	*afgp*	*chimeric*	*tomm40*
C. gobio	1	1	1	1	0	0	1
D. mawsoni	--	5	16	2	14	3	1
T. bernacchii	1	9	16	2	24	3	1
H. antarcticus	1	11	11	1	15	0	3
G. acuticeps	1	7	8	2	5	1	1
P. georgianus	1	7	7	2	15	1	1

Supplementary Figure 4 | Genes in the *afgp* locus of six notothenioid species and number of copies annotated in each assembly. Annotation includes the following genes: *afgp*: antifreeze glycoprotein genes, *tlp*: trypsinogen-like protease, *tryp1*: trypsinogen1, *tryp3*: trypsinogen3 (both *tryp1* and *tryp3* are *prss59* homologues), *tomm40:* translocase of outer mitochondrial membrane 40 homolog, *hsl*: hormone sensitive lipase (*lipeb*), *afgp/tlp*: chimeric *afgp* and *tlp* gene.

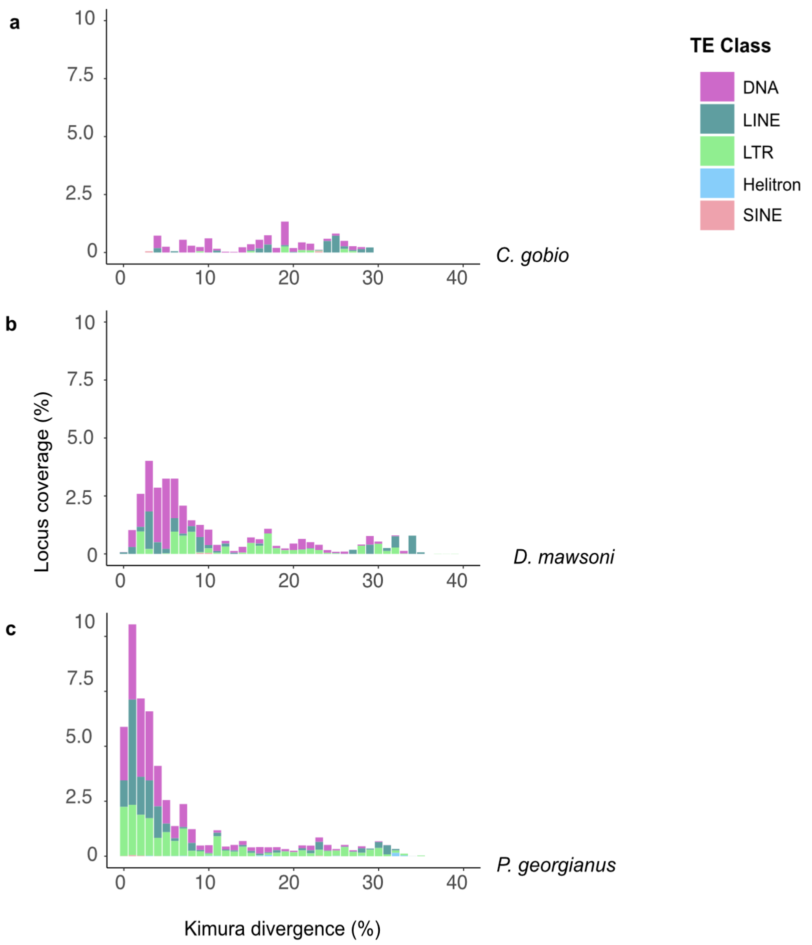

Supplementary Figure 5 | Transposon insertions in the *afgp* locus of three notothenioid species, a) *C. gobio*, b) *D. mawsoni*, and c) *P. georgianus*. Plots show distribution of TE copies based on % of divergence from their consensus sequences (x-axis), and overall coverage as % of genome (y-axis). The colours represent different TE classes. Source data are provided as Source Data file.

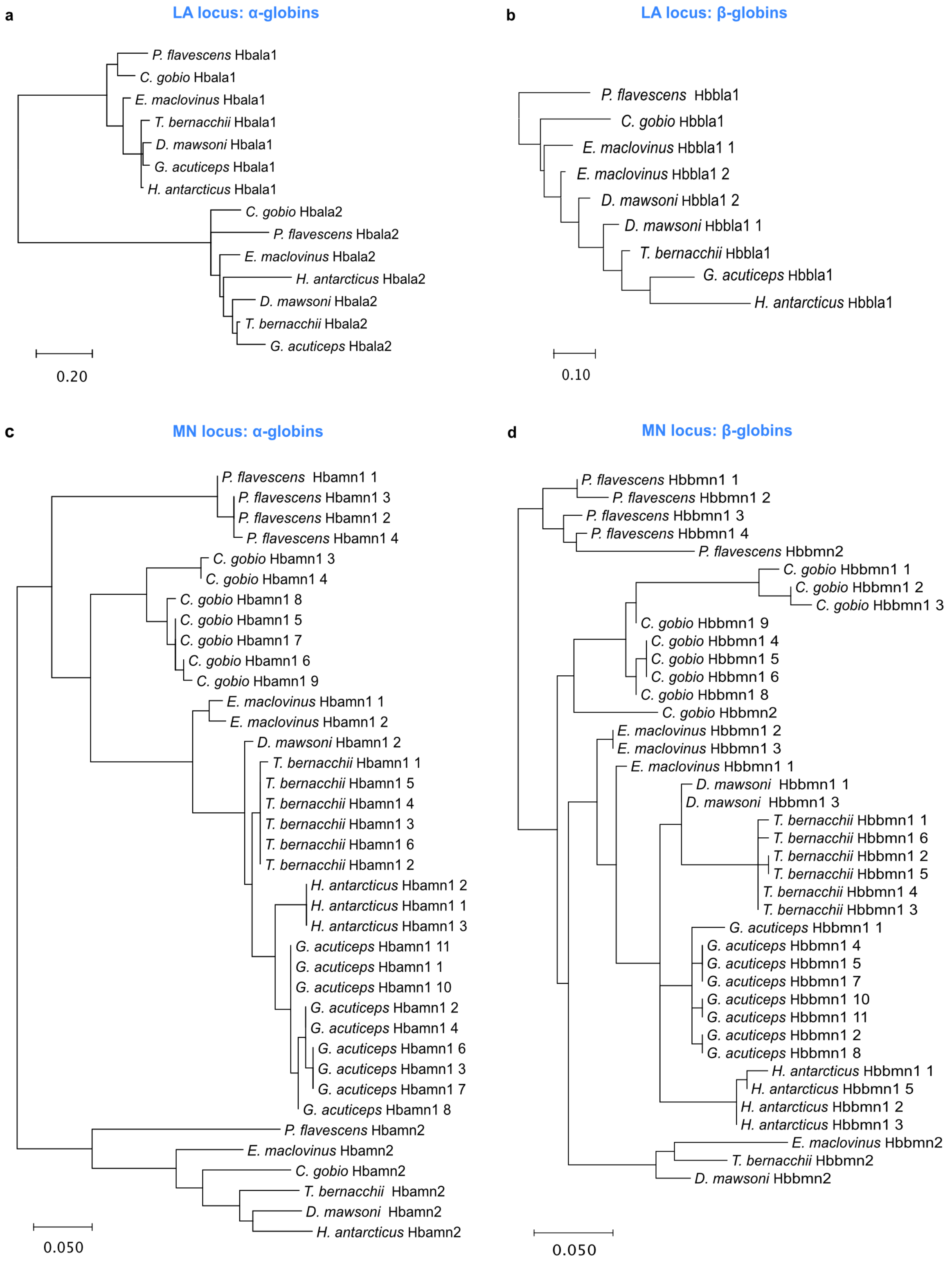

Supplementary Figure 6 | Haemoglobin protein trees (RaxML) from both clusters: a) α-globins in LA locus, b) β-globins in LA locus, c) a-globins in MN locus, d) β-globins in MN locus. The protein names are shown as Hbamn*,* Hbala for α-globins in MN and LA locus, and Hbbmn*,* Hbbla, for β-globins in MN and LA locus, followed by the number of the specific copy (for naming convention used see **Methods**). Protein alignments used to generate these trees are available in Supplementary Data 7.

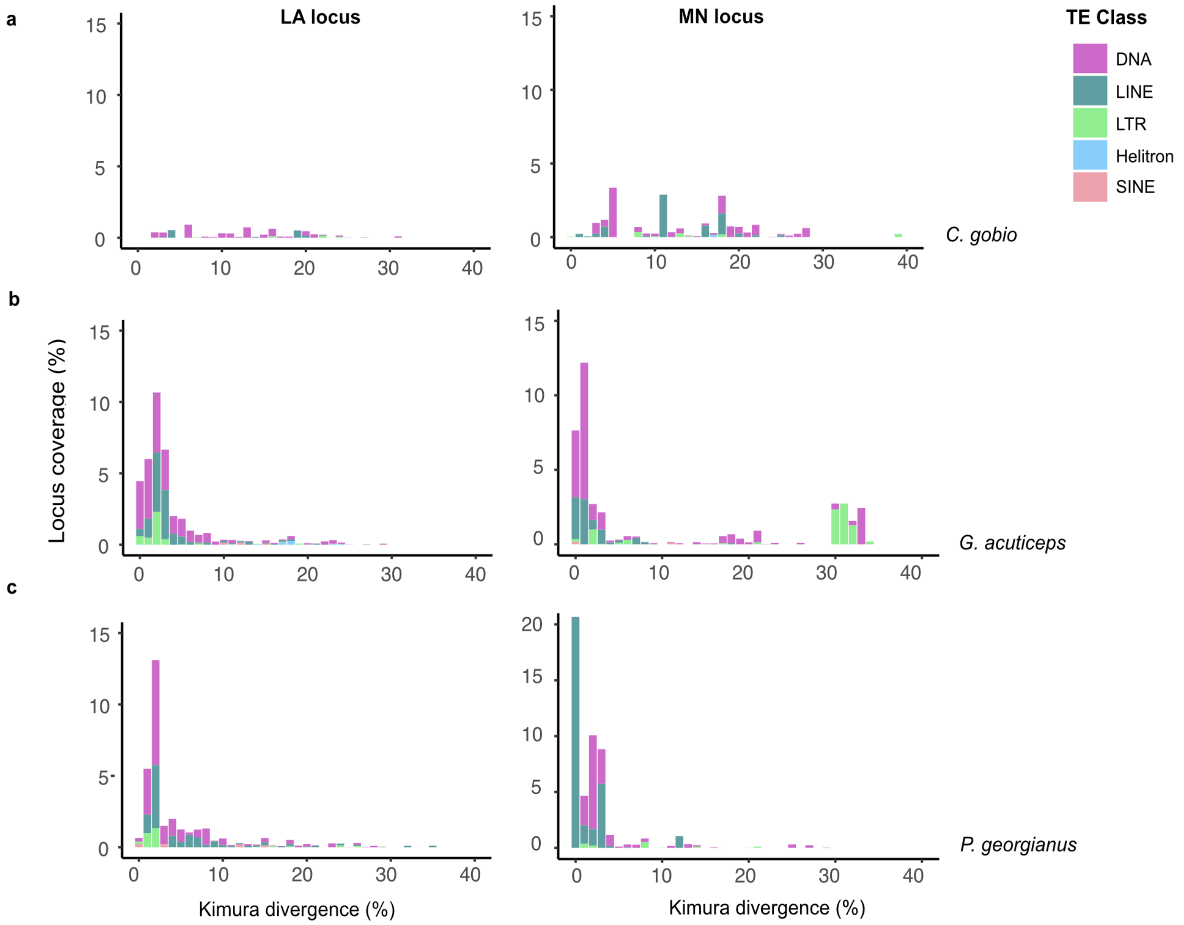

Supplementary Figure 7 | Repeat landscapes of transposon insertions in haemoglobin gene clusters LA and MN for species a) *C. gobio*, b) *G. acuticeps*, and c) *P. georgianus*. The colours represent different TE classes. Source data are provided as Source Data file.

2. SUPPLEMENTARY TABLES

Supplementary Table 1: Transposon insertions in the antifreeze gene locus. The numbers represent number of transposon copies found.

	**Species**	***C. gobio***	***D. mawsoni***	***P. georgianus***
**Class**	**Family**	**copy number**		
**DNA**	Academ-1	0	0	1
	CMC-EnSpm	**1**	**3**	**10**
	Crypton	0	2	0
	Crypton-A	0	17	13
	Crypton-H	0	-	20
	Crypton-V	1	1	4
	hAT	0	1	15
	hAT-Ac	**16**	**54**	**89**
	hAT-Blackjack	0	0	5
	hAT-Charlie	**5**	**68**	**52**
	hAT-hAT5	0	7	6
	hAT-hobo	0	0	2
	hAT-Tag1	0	1	0
	hAT-Tip100	0	15	27
	IS3EU	2	10	6
	Kolobok-E	0	0	1
	Kolobok-T2	**2**	**15**	**24**
	Maverick	**1**	**2**	**16**
	Merlin	1	0	4
	MULE-MuDR	0	0	1
	P	0	4	18
	PIF-Harbinger	**1**	**5**	**23**
	PIF-ISL2EU	0	1	2
	PiggyBac	0	5	5
	Helitrons	0	0	16
	TcMar-ISRm11	0	2	4
	TcMar-Tc1	5	3	9
	TcMar-Tc2	1	0	2
	TcMar-Tigger	0	0	1
	Zisupton	**1**	**7**	**24**
**LINE**	I	0	13	15
	L1	**2**	**5**	**18**
	L1-Tx1	0	2	0
	L2	**10**	**50**	**159**
	Penelope	0	0	5
	Proto2	0	0	4
	R2	0	4	3
	Rex-Babar	0	17	30
	RTE-BovB	1	0	13
**LTR**	RTE-X	0	2	1
	Copia	0	4	19
	DIRS	0	3	26

	ERV	0	1	2
	ERV1	0	3	26
	Gypsy	**9**	**50**	**112**
	Ngaro	0	13	42
	Pao	0	1	14
**SINE**	MIR	2	2	1
	tRNA-Core-RTE	0	0	5
	tRNA-L1	0	0	1
	tRNA-L2	0	0	1

Supplementary Table 2: Transposon insertions in haemoglobin loci: 1) MN and 2) LA. The numbers represent transposon copies.

**1) MN cluster**	**Species**	***C. gobio***	***G. acuticeps***	***P. georgianus***
**TE Class**	**TE Family**	**Number of copies**		
**DNA**	CMC-EnSpm	7	0	0
	Dada	1	0	0
	hAT-Ac	14	14	7
	hAT-Charlie	2	10	2
	hAT-Tip100	1	5	2
	Kolobok-T2	2	16	1
	MULE-MuDR	1	0	0
	PIF-ISL2EU	2	3	0
	Helitron	1	0	0
	TcMar-Tc1	4	0	0
	Academ-1	0	1	0
	Ginger-1	0	1	1
	hAT	0	1	1
	hAT-hAT5	0	2	2
	IS3EU	0	12	1
	P	0	2	3
	PIF-Harbinger	0	0	1
	Zisupton	3	0	0
**LINE**	I	1	2	1
	L2	5	28	13
	Rex-Babar	1	0	0
	RTE-BovB	3	0	0
	L1	5	0	0
**LTR**	Copia	2	0	0
	DIRS	0	1	1
	ERV1	1	0	1
	Gypsy	5	8	6
	ERV	1	0	0
	Pao	0	0	2
	Unknown	2	3	0
**SINE**	MIR	0	1	0
	tRNA-Core-RTE	0	1	0
**2) LA cluster**	**Species**	***C. gobio***	***G. acuticeps***	***P. georgianus***
**TE Class**	**TE Family**	**Number of copies**		
**DNA**	DNA	2	7	6
	CMC-EnSpm	2	4	2
	Crypton-V	0	6	0
	Crypton-A	0	0	1
	hAT	1	0	0
	hAT-Ac	14	28	21
	hAT-Charlie	2	15	9
	hAT-Tip100	1	9	3
	hAT-Blackjack	0	1	0

	hAT-hAT5	0	4	1
	hAT-hAT6	0	1	0
	IS3EU	2	2	1
	Kolobok-T2	2	4	0
	Kolobok-T3	0	0	7
	P	0	5	2
	PIF-Harbinger	1	5	5
	Helitron	0	3	0
	TcMar-Tc1	0	1	1
	Zisupton	0	1	0
	Maverick	0	0	1
	PIF-ISL2EU	0	0	1
	PiggyBac	1	0	0
	TcMar-Fot1	1	0	0
**LINE**	I	2	4	3
	L2	**3**	**34**	**31**
	Rex-Babar	1	8	7
	RTE-BovB	1	0	0
	RTE-X	1	0	0
**LTR**	ERV1	1	1	4
	Gypsy	2	9	4
	Pao	1	2	0
	Ngaro	0	0	1
	Copia	0	1	0
	ERV	0	1	0
	Unknown	1	5	4
**SINE**	SINE	0	1	1
	MIR	0	2	1
	tRNA-Core-RTE	0	0	1
	tRNA-Core-RTE	0	0	1

Supplementary Table 3: Number of interventions performed during genome curation of the five PacBio genome assemblies.

Species	*C. gobio*	*T. bernacchii*	*H. antarcticus*	*G. acuticeps*	*P. georgianus*
**Assembly version**	**fCotGob3.1**	**fTreBer1.1**	**fHarAnt1.1**	**fGymAcu1.1**	**fPseGeo1.1**
**Breaks**	9	11	1	0	29
**Joins**	114	125	161	225	387
**Inversions**	44	69	72	105	189
**Haplotig removals**	102	101	21	1569	0

Supplementary Table 4: Read statistics for the 11 Supernova assembled genome assemblies.

Species	Family	Assembly	Supernova Version	Reads (M)	Molecule length (kb)	Coverage (GenomeScope)
Akarotaxis nudiceps	Bathydraconidae	fAkaNud1	2.0.1	691	44.6	71.70
Bovichtus diacanthus	Bovichtidae	fBovDia2	2.0.0	786	71.19	54.24
Bovichtus variegatus	Bovichtidae	fBovVar2	2.0.0	718	114.77	34.99
Chaenodraco wilsoni	Channichthyidae	fChaWil1	2.0.0	686	54.96	58.68
Cryodraco antarcticus	Channichthyidae	fCryAnt1	2.0.0	743	31.17	52.50
Gobionotothen gibberifrons	Nototheniinae	fGobGib1	2.0.0	751	27.12	43.32
Histiodraco velifer	Artedidraconidae	fHisVel1	2.0.0	736	14.34	54.38
Lepidonotothen nudifrons	Nototheniinae	fLepNud1	2.0.0	738	23.83	47.27
Notothenia rossii	Nototheniinae	fNotRos1	2.0.1	790	50.25	54.14
Pagetopsis macropterus	Channichthyidae	fPagMac1	2.0.0	721	34.09	56.19
Trematomus loennbergii	Nototheniinae	fTreLoe1	2.0.1	792	37.66	46.99

Supplementary Table 5: Species used for phylogenetic analysis (Fig. 2). Accession numbers for assemblies generated in this study are listed in the Data availability section.

**Species**	**Order/Suborder**	**Family**	**Assembly**
Gasterosteus aculeatus	Gasterosteiformes	Gasterosteidae	BROAD S1
Sebastes nigrocinctus	Scorpaeniformes	Sebastidae	ASM47523v3
Perca fluviatilis	Perciformes	Percidae	GCA_003412525.1
Takifugu rubripes	Tetraodontiformes	Tetraodontidae	GCA_901000725.2
Oryzias latipes	Beloniformes	Adrianichthyidae	GCA_002234715.1
Hippocampus comes	Syngnathiformes	Syngnathidae	GCA_001891065.1
Periophthalmus magnuspinnatus	Perciformes	Gobiidae	GCA_000787105.1
Anabas testudineus	Anabantiformes	Anabantidae	fAnaTes1.2
Mastacembelus armatus	Synbranchiformes	Mastacembelidae	fMasArm1.2
Astatotilapia calliptera	Perciformes	Cichlidae	fAstCal1.2 (Rhie et al., 2021)[1]
Cottoperca gobio	Perciformes/ Notothenioidei	Bovichtidae	fCotGob3.1 (Bista et al. 2020)[2]
Bovichtus diacanthus	Perciformes/ Notothenioidei	Bovichtidae	fBovDia2 (this study)
Bovichtus variegatus	Perciformes/ Notothenioidei	Bovichtidae	fBovVar2 (this study)
Eleginops maclovinus	Perciformes/ Notothenioidei	Eleginopidae	(Chen et al., 2019)[3]
Dissostichus mawsoni	Perciformes/ Notothenioidei	Nototheniidae	(Chen et al., 2019)[3]
Pleuragramma antarctica	Perciformes/ Notothenioidei	Nototheniidae	(Baalsrud et al., 2018)[4]
Patagonotothen guntheri	Perciformes/ Notothenioidei	Nototheniidae	(Baalsrud et al., 2018)[4]
Trematomus bernacchii	Perciformes/ Notothenioidei	Nototheniidae	fTreBer1 (this study)
Gobionotothen gibberifrons	Perciformes/ Notothenioidei	Nototheniidae	fGobGib1 (this study)
Lepidonotothen nudifrons	Perciformes/ Notothenioidei	Nototheniidae	fLepNud1 (this study)
Notothenia rossii	Perciformes/ Notothenioidei	Nototheniidae	fNotRos1 (this study)
Trematomus loennbergii	Perciformes/ Notothenioidei	Nototheniidae	fTreLoe1 (this study)
Lepidonotothen larseni	Perciformes/ Notothenioidei	Nototheniidae	fLepLar1 (this study)
Lepidonotothen squamifrons	Perciformes/ Notothenioidei	Nototheniidae	fLepSqu1 (this study)
Trematomus hansoni	Perciformes/ Notothenioidei	Nototheniidae	fTreHan1 (this study)
Trematomus scotti	Perciformes/ Notothenioidei	Nototheniidae	fTreSco1 (this study)
Harpagifer kerguelensis	Perciformes/ Notothenioidei	Harpagiferidae	(Baalsrud et al., 2018)[4]
Harpagifer antarcticus	Perciformes/ Notothenioidei	Harpagiferidae	fHarAnt1 (this study)
Artedidraco skottsbergi	Perciformes/ Notothenioidei	Artedidraconidae	(Baalsrud et al., 2018)[4]
Histiodraco velifer	Perciformes/ Notothenioidei	Artedidraconidae	fHisVel1 (this study)

Dolloidraco longedorsalis	Perciformes/ Notothenioidei	Artedidraconidae	fDolLon1 (this study)
Gymnodraco acuticeps	Perciformes/ Notothenioidei	Bathydraconidae	fGymAcu1 (this study)
Akarotaxis nudiceps	Perciformes/ Notothenioidei	Bathydraconidae	fAkaNud1 (this study)
Bathydraco marri	Perciformes/ Notothenioidei	Bathydraconidae	fBatMar1 (this study)
Vomeridens infuscipinnis	Perciformes/ Notothenioidei	Bathydraconidae	fVomInf1 (this study)
Chaenocephalus aceratus	Perciformes/ Notothenioidei	Channichthyidae	JAMFTG000000000.1 (Kim et al., 2019) [5]
Pseudochaenichthys georgianus	Perciformes/ Notothenioidei	Channichthyidae	fPseGeo1 (this study)
Chaenodraco wilsoni	Perciformes/ Notothenioidei	Channichthyidae	fChaWil1 (this study)
Cryodraco antarcticus	Perciformes/ Notothenioidei	Channichthyidae	fCryAnt1 (this study)
Pagetopsis macropterus	Perciformes/ Notothenioidei	Channichthyidae	fPagMac1 (this study)
Chionobathyscus dewitti	Perciformes/ Notothenioidei	Channichthyidae	fChiDew1 (this study)

Supplementary Table 6: Tools used for genome assembly, annotation, and data analysis, including version and source.

Tool	version	source
Arrow	GenomicConsensus 2.2.2	https://github.com/PacificBiosciences/GenomicConsnsus
ASTRAL	v.5.7.3	https://doi.org/10.1186/s12859-018-2129-y
bcftools consensus	1.7	http://samtools.github.io/bcftools/bcftools.html
BEAST2	v.2.6.0	https://doi.org/10.1371/journal.pcbi.1006650
BFC		https://github.com/lh3/bfc
BLAST+	ncbi-blast-2.7.1+	ftp://ftp.ncbi.nlm.nih.gov/blast/executables/blast+/LATEST
BUSCO	v.2, v.3, v.5	https://doi.org/10.1093/bioinformatics/btv351
Bwa-mem	0.7.17-r1188	https://arxiv.org/abs/1303.3997
Canu	1.6	https://www.ncbi.nlm.nih.gov/pmc/articles/PMC5411767/
cross_genome	41873	https://sourceforge.net/projects/phusion2/files/cross_genome/
Falcon-unzip	falcon-2018.03.12-04.00	https://www.ncbi.nlm.nih.gov/pmc/articles/PMC5503144/
freebayes	v1.1.0-3-g961e5f3	https://arxiv.org/abs/1303.3997
GAP5		https://doi.org/10.1093/bioinformatics/btq268
hmmer	hmmer-3.2.1	http://hmmer.org/
IQ-TREE	v.1.7	https://doi.org/10.1093/molbev/msu300
Juicebox		https://www.ncbi.nlm.nih.gov/pmc/articles/PMC5596920/ , https://www.ncbi.nlm.nih.gov/pmc/articles/PMC6047755/
LAGAN		http://www.genome.org/cgi/doi/10.1101/gr.926603
LTR_retriever	v.2.8	https://doi.org/10.1104/pp.17.01310
MAFFT	v.7.453	https://doi.org/10.1093/molbev/mst010
MUSCLE	v.3.8.31	http://www.drive5.com/muscle
PartitionFinder	v.2.1.1	https://doi.org/10.1093/molbev/msw260
PBJelly	PBSuite_15.8.24	https://www.ncbi.nlm.nih.gov/pmc/articles/PMC3504050/
Purge Haplotigs	v1	https://www.ncbi.nlm.nih.gov/pmc/articles/PMC6267036/
Racon	https://github.com/isovic/racon.git	https://www.ncbi.nlm.nih.gov/pmc/articles/PMC5411768/
RAxML-NG		https://academic.oup.com/bioinformatics/advance-article/doi/10.1093/bioinformatics/btz305/5487384
RECON	RECON-1.08	http://eddylab.org/software/recon/
RepeatMasker	v.4.0.1	http://www.repeatmasker.org
RepeatModeler	v.2.0	http://www.pnas.org/lookup/doi/10.1073/pnas.1921046117
RepeatScout	RepeatScout-1.0.6	https://github.com/mmcco/RepeatScout
rmblast	rmblast-2.6.0+	https://www.repeatmasker.org/rmblast/
samtools		https://github.com/samtools
scaff10x	1	https://github.com/wtsi-hpag/Scaff10X
Shuffle-LAGAN		https://doi.org/10.1093/bioinformatics/btg1005
soapDeNovo2	v2	https://sourceforge.net/projects/soapdenovo2/
Solve	Solve3.2.2_08222018	https://bionanogenomics.com/downloads/bionano-solve/
Supernova	v.2.0	https://support.10xgenomics.com/de-novo-assembly/software/overview/latest/welcome
trf	TRF-4.09	https://tandem.bu.edu/trf/trf.html
wtdbg	1.1	https://doi.org/10.1038/s41592-019-0669-3

3. SUPPLEMENTARY REFERENCES

1. Rhie, A. *et al.* Towards complete and error-free genome assemblies of all vertebrate species. *Nature* **592**, 737–746 (2021).

2. Bista, I. *et al.* The genome sequence of the channel bull blenny, *Cottoperca gobio* (Günther, 1861). *Wellcome Open Res.* **5**, 148 (2020).

3. Chen, L. *et al.* The genomic basis for colonizing the freezing Southern Ocean revealed by Antarctic toothfish and Patagonian robalo genomes. *Gigascience* **8**, (2019).

4. Baalsrud, H. T. *et al.* De novo gene evolution of antifreeze glycoproteins in codfishes revealed by whole genome sequence data. *Mol. Biol. Evol.* **35**, 593–606 (2018).

5. Kim, B.-M. *et al.* Antarctic blackfin icefish genome reveals adaptations to extreme environments. *Nat. Ecol. Evol.* **3**, 469–478 (2019).
