# Supplementary material for: Genomics of cold adaptations in the Antarctic notothenioid fish radiation
Source: Nat Commun. 2023 Jun 9;14:3412. doi: 10.1038/s41467-023-38567-6 (PMC10256766; doi:10.1038/s41467-023-38567-6)
Supplement: Supplementary file 3 — Reporting Summary [file 41467_2023_38567_MOESM3_ESM.pdf]

Corresponding author(s): Iliana Bista and Richard Durbin

Last updated by author(s): 03 April 2023

# Reporting Summary

Nature Portfolio wishes to improve the reproducibility of the work that we publish. This form provides structure for consistency and transparency in reporting. For further information on Nature Portfolio policies, see our Editorial Policies and the Editorial Policy Checklist.

## Statistics

For all statistical analyses, confirm that the following items are present in the figure legend, table legend, main text, or Methods section.

| n/a | Confirmed | |
|---|---|---|
| ☐ | ☒ | The exact sample size (*n*) for each experimental group/condition, given as a discrete number and unit of measurement |
| ☐ | ☒ | A statement on whether measurements were taken from distinct samples or whether the same sample was measured repeatedly |
| ☐ | ☒ | The statistical test(s) used AND whether they are one- or two-sided<br>*Only common tests should be described solely by name; describe more complex techniques in the Methods section.* |
| ☒ | ☐ | A description of all covariates tested |
| ☒ | ☐ | A description of any assumptions or corrections, such as tests of normality and adjustment for multiple comparisons |
| ☐ | ☒ | A full description of the statistical parameters including central tendency (e.g. means) or other basic estimates (e.g. regression coefficient) AND variation (e.g. standard deviation) or associated estimates of uncertainty (e.g. confidence intervals) |
| ☐ | ☒ | For null hypothesis testing, the test statistic (e.g. *F*, *t*, *r*) with confidence intervals, effect sizes, degrees of freedom and *P* value noted<br>*Give P values as exact values whenever suitable.* |
| ☐ | ☒ | For Bayesian analysis, information on the choice of priors and Markov chain Monte Carlo settings |
| ☒ | ☐ | For hierarchical and complex designs, identification of the appropriate level for tests and full reporting of outcomes |
| ☐ | ☒ | Estimates of effect sizes (e.g. Cohen's *d*, Pearson's *r*), indicating how they were calculated |

*Our web collection on statistics for biologists contains articles on many of the points above.*

## Software and code

Policy information about availability of computer code

| | |
|---|---|
| Data collection | Sequence data collection used standard software provided by the instrument manufacturer. |
| Data analysis | Here we used sequencing data from different technologies including PacBio, Illumina HiSeq, Hi-C, and 10X Genomics to generate genome assemblies for 24 notothenioid fish species. Genome assembly approaches were different depending on the data type and appropriate tools were used (software and versions are listed below). Using these data and other additional published assemblies we performed analyses to investigate the genomic evolution of the notothenioid fish radiation. We performed phylogenetic analysis using genome wide data, to investigate the timing of the expansion of the radiation. We also performed genome-wide annotation and analysis of transposable elements and their influence in cold adaptation. Finally, we studied two gene families, the antifreeze genes, and the haemoglobin genes which were important in providing freeze avoidance (antifreeze genes), and the unique loss of haemoglobins in a subgroup of notothenioids (icefish), while also considering the effect of transposon insertions in the evolution of these genes. Below we list of the software that were used for analysis (listed as Software name, version, source):<br>Arrow, GenomicConsensus 2.2.2, https://github.com/PacificBiosciences/GenomicConsnsus<br>ASTRAL, v.5.7.3, https://doi.org/10.1186/s12859-018-2129-y<br>bcftools consensus, 1.7 ,http://samtools.github.io/bcftools/bcftools.html<br>BEAST2,  v.2.6.0, https://doi.org/10.1371/journal.pcbi.1006650<br>BFC,  https://github.com/lh3/bfc<br>BLAST+, ncbi-blast-2.7.1+, ftp://ftp.ncbi.nlm.nih.gov/blast/executables/blast+/LATEST<br>BUSCO, v.2, v.3, v.5, https://doi.org/10.1093/bioinformatics/btv351<br>Bwa-mem, 0.7.17-r1188, https://arxiv.org/abs/1303.3997<br>Canu, 1.6, https://www.ncbi.nlm.nih.gov/pmc/articles/PMC5411767/<br>cross_genome, 41873, https://sourceforge.net/projects/phusion2/files/cross_genome/ |

Falcon-unzip, falcon-2018.03.12-04.00, https://www.ncbi.nlm.nih.gov/pmc/articles/PMC5503144/
freebayes, v1.1.0-3-g961e5f3, https://arxiv.org/abs/1303.3997
GAP5, https://doi.org/10.1093/bioinformatics/btq268
hmmer, hmmer-3.2.1, http://hmmer.org/
IQ-TREE, v.1.7, https://doi.org/10.1093/molbev/msu300
Juicebox, https://www.ncbi.nlm.nih.gov/pmc/articles/PMC5596920/ , https://www.ncbi.nlm.nih.gov/pmc/articles/PMC6047755/
LAGAN, http://www.genome.org/cgi/doi/10.1101/gr.926603
LTR_retriever, v.2.8, https://doi.org/10.1104/pp.17.01310
MAFFT, v.7.453, https://doi.org/10.1093/molbev/mst010
MUSCLE, v.3.8.31, http://www.drive5.com/muscle
PartitionFinder, v.2.1.1, https://doi.org/10.1093/molbev/msw260
PBJelly, PBSuite_15.8.24, https://www.ncbi.nlm.nih.gov/pmc/articles/PMC3504050/
Purge Haplotigs, v1, https://www.ncbi.nlm.nih.gov/pmc/articles/PMC6267036/
Racon, https://github.com/isovic/racon.git https://www.ncbi.nlm.nih.gov/pmc/articles/PMC5411768/
RAxML-NG , https://academic.oup.com/bioinformatics/advance-article/doi/10.1093/bioinformatics/btz305/5487384
RECON, RECON-1.08, http://eddylab.org/software/recon/
RepeatMasker, v.4.0.1, http://www.repeatmasker.org
RepeatModeler, v.2.0, http://www.pnas.org/lookup/doi/10.1073/pnas.1921046117
RepeatScout, RepeatScout-1.0.6, https://github.com/mmcco/RepeatScout
rmblast, rmblast-2.6.0+, https://www.repeatmasker.org/rmblast/
samtools, https://github.com/samtools
scaff10x, 1, https://github.com/wtsi-hpag/Scaff10X
Shuffle-LAGAN, https://doi.org/10.1093/bioinformatics/btg1005
soapDeNovo2, v2, https://sourceforge.net/projects/soapdenovo2/
Solve, Solve3.2.2_08222018, https://bionanogenomics.com/downloads/bionano-solve/
Supernova, v.2.0, https://support.10xgenomics.com/de-novo-assembly/software/overview/latest/welcome
trf, TRF-4.09, https://tandem.bu.edu/trf/trf.html
wtdbg, 1.1, https://doi.org/10.1038/s41592-019-0669-3

For manuscripts utilizing custom algorithms or software that are central to the research but not yet described in published literature, software must be made available to editors and reviewers. We strongly encourage code deposition in a community repository (e.g. GitHub). See the Nature Portfolio guidelines for submitting code & software for further information.

# Data

Policy information about availability of data

All manuscripts must include a data availability statement. This statement should provide the following information, where applicable:

- Accession codes, unique identifiers, or web links for publicly available datasets
- A description of any restrictions on data availability
- For clinical datasets or third party data, please ensure that the statement adheres to our policy

The genome assemblies generated in this study have been deposited on NCBI under BioProject PRJEB53202 and the following accessions:
C. gobio: GCA_900634415.1[https://www.ncbi.nlm.nih.gov/assembly/GCA_900634415.1/] (alt. hap. GCA_900634435.1[https://www.ncbi.nlm.nih.gov/assembly/GCA_900634435.1/]),
T. bernacchii GCA_902827165.1[https://www.ncbi.nlm.nih.gov/assembly/GCA_902827165.1/] (alt. hap. GCA_902827105.1[https://www.ncbi.nlm.nih.gov/assembly/GCA_902827105.1/]),
H. antarcticus GCA_902827135.1[ttps://www.ncbi.nlm.nih.gov/assembly/GCA_902827135.1/] (alt. hap. GCA_902827095.1[https://www.ncbi.nlm.nih.gov/assembly/GCA_902827095.1/]),
G. acuticeps GCA_902827175.1[https://www.ncbi.nlm.nih.gov/assembly/GCA_902827175.1/] (alt. hap. GCA_902827185.1[https://www.ncbi.nlm.nih.gov/assembly/GCA_902827185.1/]),
P. georgianus GCF_902827115.1[https://www.ncbi.nlm.nih.gov/assembly/GCF_902827115.1/]
and GCA_902827115.2[ttps://www.ncbi.nlm.nih.gov/assembly/GCA_902827115.2/] (alt. hap GCA_902827155.1[https://www.ncbi.nlm.nih.gov/assembly/GCA_902827155.1/]),
B. diacanthus GCA_943590825.1[https://www.ncbi.nlm.nih.gov/assembly/GCA_943590825.1/],
B. variegatus GCA_943593645.1[https://www.ncbi.nlm.nih.gov/assembly/GCA_943593645.1/],
T. loennbergii GCA_943590855.1[https://www.ncbi.nlm.nih.gov/assembly/GCA_943590855.1/],
L. larseni GCA_943594155.1[https://www.ncbi.nlm.nih.gov/assembly/GCA_943594155.1/],
L. squamifrons GCA_943593335.1[https://www.ncbi.nlm.nih.gov/assembly/GCA_943593335.1/],
T. hansoni GCA_943593355.1[https://www.ncbi.nlm.nih.gov/assembly/GCA_943593355.1/],
T. scotti GCA_943590805.1[https://www.ncbi.nlm.nih.gov/assembly/GCA_943590805.1/],
L. nudifrons GCA_943590975.1[https://www.ncbi.nlm.nih.gov/assembly/GCA_943590975.1/],
G. gibberifrons GCA_943591055.1[https://www.ncbi.nlm.nih.gov/assembly/GCA_943591055.1/],
N. rossii GCA_943590865.1[https://www.ncbi.nlm.nih.gov/assembly/GCA_943590865.1/],
D. longedorsalis GCA_943591025.1[https://www.ncbi.nlm.nih.gov/assembly/GCA_943591025.1/],
H. velifer GCA_943590885.1[https://www.ncbi.nlm.nih.gov/assembly/GCA_943590885.1/],
A. nudiceps GCA_943590845.1[https://www.ncbi.nlm.nih.gov/assembly/GCA_943590845.1/],
B. marri GCA_943591095.1[https://www.ncbi.nlm.nih.gov/assembly/GCA_943591095.1/],
V. infuscipinnis GCA_943590875.1[https://www.ncbi.nlm.nih.gov/assembly/GCA_943590875.1/],
C. wilsoni GCA_943593825.1[https://www.ncbi.nlm.nih.gov/assembly/GCA_943593825.1/],
C. antarcticus GCA_943590835.1[https://www.ncbi.nlm.nih.gov/assembly/GCA_943590835.1/],
P. macropterus GCA_943590895.1[https://www.ncbi.nlm.nih.gov/assembly/GCA_943590895.1/],
C. dewitti GCA_943594065.1[https://www.ncbi.nlm.nih.gov/assembly/GCA_943594065.1/].
Gene annotation for species C. gobio is available on Ensembl [www.ensembl.org], and for T. bernacchii, H. antarcticus, G. acuticeps, P. georgianus gene annotations are available on Ensembl Rapid Release [https://rapid.ensembl.org/]. RefSeq annotations for C. gobio, T. bernacchii, G. acuticeps, and P. georgianus can be found on

NCBI under assembly accession numbers. All raw sequencing data are available on NCBI (accessions listed in Supplementary Data 1). Data used for phylogenetic analysis of notothenioid and outgroup fish species, along with alignments and phylogenetic trees are available on Dryad: https://doi.org/10.5061/dryad.80gb5mktn.

## Human research participants

Policy information about studies involving human research participants and Sex and Gender in Research.

| | |
|---|---|
| Reporting on sex and gender | N/A |
| Population characteristics | N/A |
| Recruitment | N/A |
| Ethics oversight | N/A |

Note that full information on the approval of the study protocol must also be provided in the manuscript.

# Field-specific reporting

Please select the one below that is the best fit for your research. If you are not sure, read the appropriate sections before making your selection.

☒ Life sciences ☐ Behavioural & social sciences ☐ Ecological, evolutionary & environmental sciences

For a reference copy of the document with all sections, see nature.com/documents/nr-reporting-summary-flat.pdf

# Life sciences study design

All studies must disclose on these points even when the disclosure is negative.

| | |
|---|---|
| Sample size | We generated genome assemblies for 24 species and used additional published data to supplement analysis where needed. Due to the nature of the analysis and the availability of appropriately preserved tissue samples needed for each type of sequencing we used sequencing technologies as follows: PacBio hybrid for five species, 10X Genomics for 11 species, and Illumina HiSeq for 8 species. A single specimen was used for data generation as this is the best practice for sequencing, with the exception of species (C. gobio) where a second individual was used for generation of HiC data (use of other specimen for this type of data does not impact assembly quality). The above technical requirements coupled with specimen availability determined our sample size. |
| Data exclusions | Only sequence data that failed quality control were excluded or repeated. |
| Replication | Analysis was performed independently for different species requiring genome assembly. To finalize each genome assembly we performed manual curation to verify correctness. For phylogenetic analyses we used Bayesian and bootstrap methods were used to test the robustness of phylogenetic inferences. All data used for analyses have been submitted to public databases as described in data availability section. |
| Randomization | Randomization was used when appropriate, as in phylogenetic analysis through the use of bootstrap methods. |
| Blinding | This study did not use any blinding during analysis, which is not required for approaches used. |

# Reporting for specific materials, systems and methods

We require information from authors about some types of materials, experimental systems and methods used in many studies. Here, indicate whether each material, system or method listed is relevant to your study. If you are not sure if a list item applies to your research, read the appropriate section before selecting a response.

## Materials & experimental systems

| n/a | Involved in the study | |
|---|---|---|
| ☒ | ☐ | Antibodies |
| ☒ | ☐ | Eukaryotic cell lines |
| ☒ | ☐ | Palaeontology and archaeology |
| ☐ | ☒ | Animals and other organisms |
| ☒ | ☐ | Clinical data |
| ☒ | ☐ | Dual use research of concern |

## Methods

| n/a | Involved in the study | |
|---|---|---|
| ☒ | ☐ | ChIP-seq |
| ☒ | ☐ | Flow cytometry |
| ☒ | ☐ | MRI-based neuroimaging |

## Animals and other research organisms

Policy information about studies involving animals; ARRIVE guidelines recommended for reporting animal research, and Sex and Gender in Research

| | |
|---|---|
| Laboratory animals | No laboratory animals were used in this study. |
| Wild animals | Samples of the 24 species for subsequent sequencing were collected from wild fish caught during research studies, killed using approved procedures, with approved permits of the source institutions and local authorities involved. The persons who collected the samples, georgaphic location, sex, methods of collection and preservation are listed in Supplementary Table 1. |
| Reporting on sex | Sex was determined by morphology or histology. The sex is determined as supplementary information, whilst also reporting cases where determining the sex was not possible. Our findings are not dependent on sex. |
| Field-collected samples | No laboratory work was conducted on field collected animals. |
| Ethics oversight | Any procedures and sampling undertaken in relation to this work were performed in compliance with all relevant ethical regulations.<br>For samples provided by TD and JHP, all procedures on animals were performed following IACUC protocol #13-27RRAA approved by the University of Oregon, and access to Antarctic Specially Protected Areas 152 (Western Bransfield Strait) and 153 (Eastern Dallmann Bay) for fishing was authorized by the Antarctic Conservation Act Permit ACA 2016-025.<br>Fish collection and sampling by C-HC Cheng complied with animal use protocol #12123 approved by the Institutional Animal Care and Use Committee (IACUC), University of Illinois Urbana-Champaign. Specimens from Antarctic Special Protected Areas ASPA152 and ASPA153 were collected under the ACA (Antarctic Conservation Act) permit number 2014-030<br>For samples provided by MSC British Antarctic Survey, all procedures followed UK animal welfare regulations and specimen euthanasia procedures under Schedule 1.<br>For samples provided by HWD collection and sampling of notothenioid fishes between 2004 and 2018 was approved under Northeastern University Institutional Animal Care and Use Committee protocols (03-0314R, 08-0101R, 09-0308R, 10-1235R, 12-0306R, 15-0207R, 18-0103R). Specimens from Antarctic Special Protected Areas ASPA152 and ASPA153 were collected by HWD under Antarctic Conservation Act permit numbers 2009-1, 2012-014, 2013-028, 2014-029, and 2016-025. Collection of notothenioids by HWD at the Falkland Islands/Islas Malvinas was permitted by Argentina and the United Kingdom, sampling at Bouvetøya Island was approved by Norway, and fishing at Tristan da Cunha was permitted by the United Kingdom. |

Note that full information on the approval of the study protocol must also be provided in the manuscript.
