# Supplementary material for: Genomics of cold adaptations in the Antarctic notothenioid fish radiation
Source: Nat Commun. 2023 Jun 9;14:3412. doi: 10.1038/s41467-023-38567-6 (PMC10256766; doi:10.1038/s41467-023-38567-6)
Supplement: Supplementary file 4 — Description of Additional Supplementary Files [file 41467_2023_38567_MOESM4_ESM.docx]

**Description of Additional Supplementary Files**

File Name: Supplementary Data 1

Description: List of species used for analysis. The list represents taxonomic information for target species, assembly type, tissue type used for sequencing, tissue preservation, method used for DNA/RNA extraction, specimen collection information, biosample IDs and raw data accessions. WAP: West Antarctic Peninsula. NA: not available.

File Name: Supplementary Data 2

Description: Species sequenced, and data types used. Assembly statistics and accession information.

File Name: Supplementary Data 3

Description: Assembly statistics for SoapDeNovo assembled genomes. The primary assembly version (Soap) was scaffolded using synteny (Synteny) with the nearest PacBio genome (SyntenyWith).

File Name: Supplementary Data 4

Description: Number of genes identified during Ensembl annotation process for five assemblies (NC; non-coding).

File Name: Supplementary Data 5

Description: Composition of transposable elements in assembled genomes.

File Name: Supplementary Data 6

Description: Metrics of PacBio sequencing data.

File Name: Supplementary Data 7

Description: Protein alignments used to generate phylogenetic trees in Supplementary Fig.6
